# Temporary Survival Increasing the Diversity of Culturable Heterotrophic Bacteria in the Newly Exposed Moraine at a Glacier Snout

**DOI:** 10.3390/biology11111555

**Published:** 2022-10-24

**Authors:** Yang Liu, Yeteng Xu, Xiaowen Cui, Binglin Zhang, Xinyue Wang, Xiang Qin, Jinxiu Wang, Yanzhao Li, Wei Zhang, Guangxiu Liu, Tuo Chen, Gaosen Zhang

**Affiliations:** 1 State Key Laboratory of Cryospheric Sciences, Northwest Institute of Eco-Environment and Resources, Chinese Academy of Sciences, Lanzhou 730000, China; 2 University of Chinese Academy of Sciences, No. 19A Yuquan Road, Beijing 100049, China; 3 Key Laboratory of Extreme Environmental Microbial Resources and Engineering, Lanzhou 730000, China; 4 Key Laboratory of Desert and Desertification, Northwest Institute of Eco-Environment and Resources, Chinese Academy of Sciences, Lanzhou 730000, China; 5 College of Geography and Environment Science, Northwest Normal University, Lanzhou 730070, China; 6 School of Stomatology, Lanzhou University, Lanzhou 730000, China

**Keywords:** culturable, heterotrophic bacteria, temporary survival, extremophiles, glacier snout, Laohugou Glacier No. 12

## Abstract

**Simple Summary:**

Adaptative extremophiles are frequently found in various glacial ecological niches, such as glacial meltwater, ice, snow, and permafrost. However, no systematic study has investigated the diverse and temporary survival of culturable bacteria in newly exposed moraines around glacier snouts. The findings of this study revealed the diversity of culturable heterotrophic bacteria in a newly exposed moraine and demonstrated the evolution, competition, and selective growth of bacteria facing primary succession. This study not only helps to understand the high diversity of culturable bacteria in the newly exposed moraine at a glacier snout but also provides a theoretical basis for the study of microbial resources surviving in the transition region between glaciers and retreats.

**Abstract:**

Laohugou Glacier No. 12 is located on the northern slope of the western Qilian Mountains with a temperate continental wet climate and an extremely cold winter. Bacteria in a newly exposed moraine have to cope with various pressures owing to deglaciation at the glacier snout. However, limited information is available regarding the high diversity and temporary survival of culturable heterotrophic bacteria under various environmental stresses. To examine the tolerance of extremophiles against varying environmental conditions in a newly exposed moraine, we simulated environmental stress in bacterial cultures. The results showed that the isolated strains belonged to actinobacteria, Proteobacteria, Bacteroidetes, Deinococcus-Thermus, and Firmicutes. Actinobacteria was the most abundant phylum, followed by Proteobacteria, at both high and low temperatures. *Pseudarthrobacter* was the most abundant genus, accounting for 14.2% of the total isolates. Although several microorganisms grew at 10 °C, the proportion of microorganisms that grew at 25 °C was substantially higher. In particular, 50% of all bacterial isolates grew only at a high temperature (HT), whereas 21.4% of the isolates grew at a low temperature (LT), and 38.6% of the isolates grew at both HT and LT. In addition, many radiation-resistant extremophiles were identified, which adapted to both cold and oxidative conditions. The nearest neighbors of approximately >90% of bacteria belonged to a nonglacial environment, such as oil-contaminated soil, rocks, and black sand, instead of glacial niches. This study provides insights into the ecological traits, stress responses, and temporary survival of culturable heterotrophic bacteria in a newly exposed moraine with variable environmental conditions and the relationship of these communities with the non-glacial environment. This study may help to understand the evolution, competition, and selective growth of bacteria in the transition regions between glaciers and retreats in the context of glacier melting and retreat owing to global warming.

## 1. Introduction

Owing to global warming, deglaciation has been occurring worldwide for the past 100 years [1]. The retreat of glaciers leads to the exposure of soil and rocks that were once covered in ice [2,3]. The forefield environment formed owing to glacial retreat represents a natural succession of a chronosequence of physical, chemical, and biological gradients [4], which can be used as a natural experiment for assessing the ecological and evolutionary response of microbial communities to fluctuations in environmental conditions [3,5,6]. Bacteria present in glacial ice are the initial colonizers of most primary succession environments, followed by fungi and algae, especially in high-latitude/high-altitude glacial retreat regions [7,8]. Therefore, the study of bacterial communities and their survival strategies in glacial retreat regions has received significant attention. Bacterial succession in glacial retreat regions has been investigated under differing climatic conditions and with various soil features with complete chronosequences [5,9,10]. Bhattacharya et al. reported that specialized functions of bacterial communities that inhabit retreat regions may result in nutrient inputs to soil and vegetation development, thus providing feedback to climate change [10]. The soil age of retreat regions is influenced by base saturation, pH, soil C and N contents, and plant coverage, with similar influences, also related to the bacteria succession along the forefield [5,11]. Moreover, sediments beneath the ice of the glacier terminus have both microoxic and anoxic conditions owing to the presence of bacteria with both aerobic and anaerobic metabolic mechanisms [12]. To date, few studies have reported on bacteria inhabiting newly formed moraine exposed between old retreat soil and dark soil beneath the ice at glacier snouts.

Increasing temperatures owing to global warming accelerate the melting of ice at glacier snouts [13,14]. Newly exposed moraine is disrupted owing to dramatic changes in the ecological environment [15,16], and the ecological niches of the original bacteria are disturbed owing to various environmental stresses, which may constrain the growth of bacteria [6,11,17]. The altered temperature is detrimental to the growth of cold-adapted strains resident beneath the ice [13]. Complete exposure transforms the microoxic and anoxic environments into an oxic ecological environment [12]. Additionally, changes in UV irradiation and nutrition affect the bacterial community in the newly exposed moraine [6]. Bacterial communities are highly diverse in the melting areas at glacier snouts [6,17]. Bacteria exposed to newly formed moraines compete with resident bacteria for ecological niches because glacial retreat reduces the ecological resources required for the growth of cold-adapted bacteria [6,18], and resident bacteria are sensitive to newly exposed moraines with dramatic environmental changes [12,19]. Although either resident or cold-adapted bacteria can survive in such an environment, the resident bacteria may prefer the original cold niches for growth [13]. Ecologists have labeled such bacteria as “opportunists”, which can rapidly respond to and dominate after a disturbance event. A higher proportion of opportunists can survive in the youngest soil [4,20]. Plant, eolian deposition, precipitation events, glacial dust, and aerosol can be the original ecological niches of such opportunists [4,5,21]. Therefore, we speculated that the real “seeds pool”, containing all viable microorganisms at the glacier snout, is much bigger than what the environment selects, and bacteria in moraines cannot adapt to environmental changes for growth stably but can temporarily survive here. The presence of such bacteria may activate and enhance more features of microbial communities and accelerate the transformation of retreat soil from its original status as a carbon sink into a carbon source [19,22,23].

Laohugou Glacier No. 12 is the largest continental glacier originating in the Qilian Mountains [24], with a low annual mean temperature of −11.8 °C over the past decade, which is always higher than the surface temperature of the glacier (the meteorological data were obtained from an automatic weather station (AWS) on the Laohugou glacier). Zhang et al. suggested that the increasing global temperature is the key factor for the retreat of the glacier and affects the stability of microbial communities [18]. Therefore, examining the bacterial communities in the newly retreating moraine of the Laohugou glacier snout can help to understand the patterns of biodiversity in melting glaciers at high elevations [6,25]. The survival traits of bacteria living in such an extreme environment remain unknown. The cultivation and characterization of extremophiles are important methods for understanding the diversity of culturable bacteria and assessing the mechanisms underlying bacterial growth and resistance or tolerance in extreme ecological environments [26]. In this study, we examined the original ecological niches and tolerance mechanisms of extremophiles in the newly exposed moraine with variable environmental conditions. We stimulated bacterial isolates with both low and high temperatures and cultured them in various oligotrophic media with different pretreatments of oxidation and irradiation to simulate environmental stress. This study aimed to assess (1) the diversity of heterotrophic bacteria with rapid adaptation to temperature changes in an extremely cold and high-elevation glacier snout, (2) the growth of the isolated bacteria at different temperatures, (3) the effects of environmental changes on the diversity and temporary survival of the isolated bacteria, (4) the radiation resistance in the isolated bacteria, and (5) the taxonomic novelty for ecological cultivation strategies. This study not only provides a comprehensive overview of bacterial diversity in the newly exposed moraine at the glacier snout of Laohugou Glacier No. 12 but also describes the glacier snout as a common habitat for radiation-resistant extremophiles during glacial retreat.

## 2. Materials and Methods

### 2.1. Site Description and Sample Collection

**Figure 1:**
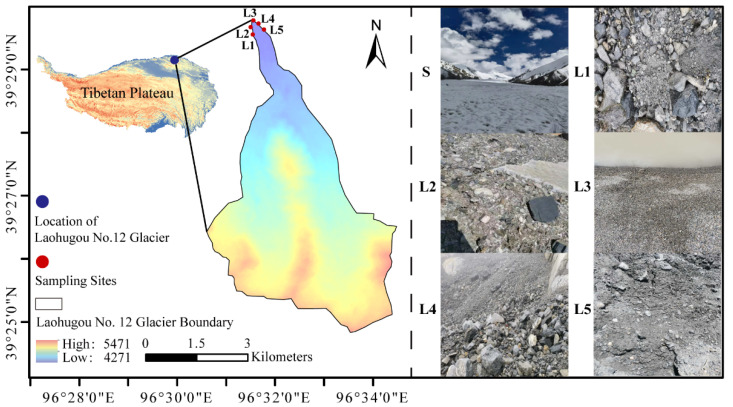
Location of Laohugou Glacier No. 12: Overall view of the glacier (S), and sampling sites on the moraine (L1–L5).

Laohugou Glacier No. 12 (Figure 1) is located on the northern slope of the western Qilian Mountains (96°31′ E, 39°30′ N), which are located on the northeastern edge of the Tibetan Plateau. It is the largest glacier in the Laohugou basin (with an altitude of 4000–5010 m above sea level (a.s.l.)) [25,27]. The study region has a temperate continental wet climate, with a cold wet summer and an extremely cold snowy winter. The annual mean temperature was −5.3 °C (range, −25.0–10.8 °C) in 2021; the minimum and maximum temperatures were −25.7 °C and 10.8 °C, respectively, from 2017 to 2021; and the mean annual precipitation is 358.6 mm (the meteorological data were obtained from an AWS on the Laohugou glacier). 

A total of 15 soil samples were collected from the sites L1–L5 along an altitude of 4273–4300 m a.s.l. near the glacier snout in April 2021. Three samples (approximately 500 g) were collected at a soil depth of 0–50 mm from each site, sealed in sterile bags (Labplas, EFR-5590E, TWIRL’EM^®^, Sainte-Julie, QC, Canada), and temporarily stored on ice in the field. The samples were immediately transferred to the laboratory and stored at −20 °C until further use. 

### 2.2. Analysis of the Physicochemical Properties of the Moraine

All soil samples were analyzed for electrical conductivity (EC), pH, water content (WC), total nitrogen (TN), total organic carbon (TOC), inorganic carbon (IC), K^+^, Ca^2+^, Na^+^, and Mg^2+^. To assess soil salinity, EC was measured using fresh soil and water in a ratio of 1:5 (*w*/*v*) using a conductivity meter (DDSJ-308A, Leici, Shanghai, China). The pH was measured using fresh soil and water in a ratio of 1:2.5 (*w*/*v*) using a pH meter (PT-10, Sartorius, Göttingen, Germany). WC was calculated based on the D-value (difference in moisture content before and after drying), percentage of dry weight, and aluminum boxes with 20 g of fresh soil samples were dried for 24 h at 105 °C. The content of TN, IC, and TOC was measured using an element analyzer (Elementar Analyse system GmbH, Hanau, Germany). The concentration of K^+^, Ca^2+^, Na^+^, and Mg^2+^ ions was determined via atomic absorption spectroscopy (AAS) (Thermo Fisher Scientific, Waltham, MA, USA). Each sample was measured thrice. All meteorological data, including temperature and irradiation, were obtained from an AWS on the Laohugou glacier and the ecological environment comprehensive observation and research station, Chinese Academy of Sciences, in November 2021.

### 2.3. Isolation of Culturable Heterotrophic Bacteria using Distinct Culture Strategies

Four types of media were used for the isolation and enumeration of bacteria (Table S1): Reasoner’s 2A agar (R2A), peptone–yeast–glucose–vitamin agar (PYGV), tryptic soy agar (TSA), and 10-fold-diluted tryptic soy agar (TSA 0.1x). The detailed culture strategies are as follows: (1) Approximately 5 g of soil samples was mixed with 10 mL of sterile saline solution (0.85% NaCl in distilled water) and sterile glass beads in a sterile flask. (2) All samples were divided into three parts; one part was used for normal culture, whereas the other two parts were respectively irradiated with UV-C at the dose of 100 J/m^2^ (after being plated) or treated with H_2_O_2_ at a final concentration of 10 mM in a sterile flask with sterile glass beads. (3) After the pre-treatment, the mixed bacterial suspension from each sample was placed in a shaker incubator at 30 °C and 200 rpm for 40 min. (4) The suspensions were diluted serially, and 100 μL of the dilutions (10^−3^, 10^−4^, and 10^−5^) were plated onto agar plates (with the four abovementioned agar media) (Table S1). (5) All plates were incubated at a low temperature of 10 °C (LT) and a high temperature of 25 °C (HT) for 1 month. For each treatment with different dilutions as mentioned above, bacterial colonies grown on at least three agar media plates were counted to meet the minimum number for statistical analyses.

After the colonies were formed, isolates were purified by streaking on the same agar plates, which were incubated at the same temperature. The pure cultures of the isolated strains were maintained at −80 °C in a medium supplemented with 20% glycerol (*v*/*v*). 

### 2.4. Identification of Bacterial Isolates through 16S rRNA Gene Sequencing

The total genomic DNA of bacterial isolates was extracted using the Bacterial DNA Extraction Kit (Omega Bio-Tek, Norcross, GA, USA) according to its manufacturer’s instructions. The 16S rRNA gene fragments of the isolates were amplified using the primers 27F and 1492R [28] with PCR conditions as described previously [26]. Amplicons were purified and sent to Qinke (Qinke company, Xi’an, China) for sequencing on an Applied Biosystems 3730XL sequencer (ABI 3730XL). The MEGA 11 tool was used to assemble the whole 16S rRNA gene sequences [29]. BLAST analysis of the 16S rRNA gene sequences using the EzTaxon database (EzBioCloud.net | Search about Bacteria or Archaea) was performed to obtain the top hits (described species) with type material sequencings [30]. The threshold value of 98.7% for 16S rRNA gene similarity was used to assess whether the isolates were potential new species [31]. 

### 2.5. Growth Temperature Range and Radiation Resistance 

To evaluate the tolerated growth temperature range of each strain, cell suspensions of all strains were prepared by incubating the samples with the optimal medium in a shaker incubator at 200 rpm and 5, 10, 20, 25, 30, 35, 40, 45, and 50 °C. OD_600_ values were estimated to assess the growth of cells at different temperatures. 

To assess the radiation-resisting ability of extremophiles, cell suspensions of all strains were prepared as described above. Pure cultures were grown in media to log phase, and the cell suspensions were adjusted to an OD_600_ of 1. The suspensions were serially diluted (10^3^–10^4^ folds) and plated on their original agar plate (the agar medium used to isolate the bacteria) [32]. We selected the reference type strains *Deinococcus radiodurans* R1 DSM 20539^T^ and *Escherichia coli* BL 21 DSM 30083^T^ [33] (German collection of microorganisms and cell cultures (DSMZ)) as the positive and negative controls, respectively. After 7–14 days of incubation, colony-forming units (CFUs) were counted to calculate the survival rate (SR) to assess the ability of bacteria to develop non-ionizing (UV-C) resistance. Three independent tests were performed to meet the minimum number for statistical analyses.

### 2.6. Data Analysis

The map of sampling sites was drawn using ArcGIS (version 10.5). The R software (version 3.6.1) was used to perform a Spearman analysis to assess the correlation between environmental factors and the abundance of each bacterial species and to perform a one-way analysis of variance (ANOVA). The Cytoscape software (version 3.7.0) was used to visualize the correlation networks [34]. The relationship between soil properties and the abundance of culturable bacteria was determined via redundancy analysis (RDA) using CANOCO (version 4.5, Plant Research International, Wageningen, Netherlands), and the significance level was assessed based on F- and *p*-values [35]. Origin Pro 2022 was used to draw figures and analyze data. 

## 3. Results

### 3.1. Physicochemical Properties of the Moraine 

**Figure 2:**
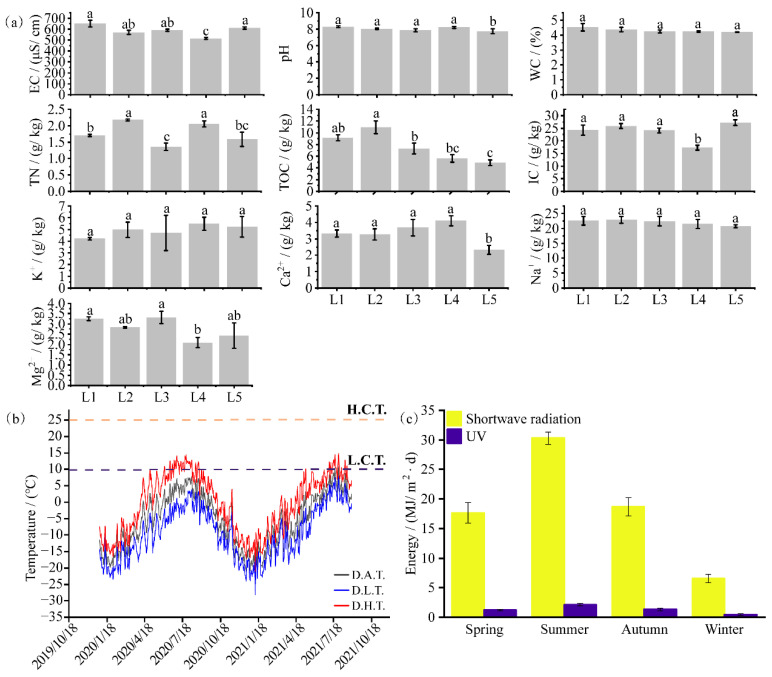
Characteristics of environmental factors in the study region. (**a**) Physicochemical properties of soil samples collected from the moraine. (**b**) Temperature changes. D.A.T., D.L.T., and D.H.T. represent the daily average temperature, daily lowest temperature, and daily highest temperature, respectively. H.C.T. and L.C.T. represent the highest culture temperature and the lowest culture temperature, respectively. (**c**) Characteristics of seasonal radiation changes in the study region. Different letters over the bars in (**a**) indicate significant differences in Physicochemical indicators at different sites.

As shown in Figure 2a, the EC of soil samples varied between 514 and 651 μS/cm, pH ranged from 7.73 to 8.28, and WC ranged from 4.19 to 4.52%. The concentration of TN was higher in samples collected from sites L2 and L4 (2.18 and 2.05 g/kg, respectively) than in those collected from sites L1, L3, and L5. Samples collected from sites L1 and L2 had higher concentrations of TOC (9 g/kg), and the concentration of IC at these sites varied between 17.35 and 27.20 g/kg. The concentration of K^+^ and Na^+^ ions was not significantly different among samples collected from the five sites. The concentration of Ca^2+^ ions was lowest in samples collected from site L5 (2.34 g/kg), whereas that in samples collected from the other four sites ranged from 3.31 to 4.10 g/kg. Significant differences were observed in the concentration of Mg^2+^ ions among samples collected from sites L1–L5 (2.09–3.25 g/kg) (Figure 2a).

### 3.2. Meteorological Characteristics of the Study Region 

According to the data obtained from an AWS, the average daily temperature of the study region exceeded 0 °C on one-third of the days per year from 2016 to 2021. The highest and lowest daily temperatures were 14.83 °C and −28.10 °C, respectively, in the past 2 years (Figure 2b). There were only a few days with the highest daily temperature of >10 °C, and only three days (28–30 August 2021) had an average daily temperature of >10 °C in the past 2 years (Figure 2b). In addition to low temperatures, higher shortwave radiation was common in the study region. During summer, the study region received the highest average daily shortwave and UV radiations of 30.27 MJ/m^2^·d and 2.12 MJ/m^2^·d, respectively. Similar shortwave radiation was observed in spring and autumn; however, UV radiation was higher in autumn (1.30 MJ/m^2^·d) than in spring (Figure 2c). Moreover, shortwave and UV radiations were lowest in winter.

### 3.3. Abundance of Heterotrophic Bacteria and Phylogenetic Diversity of Strains at HT and LT

**Figure 3:**
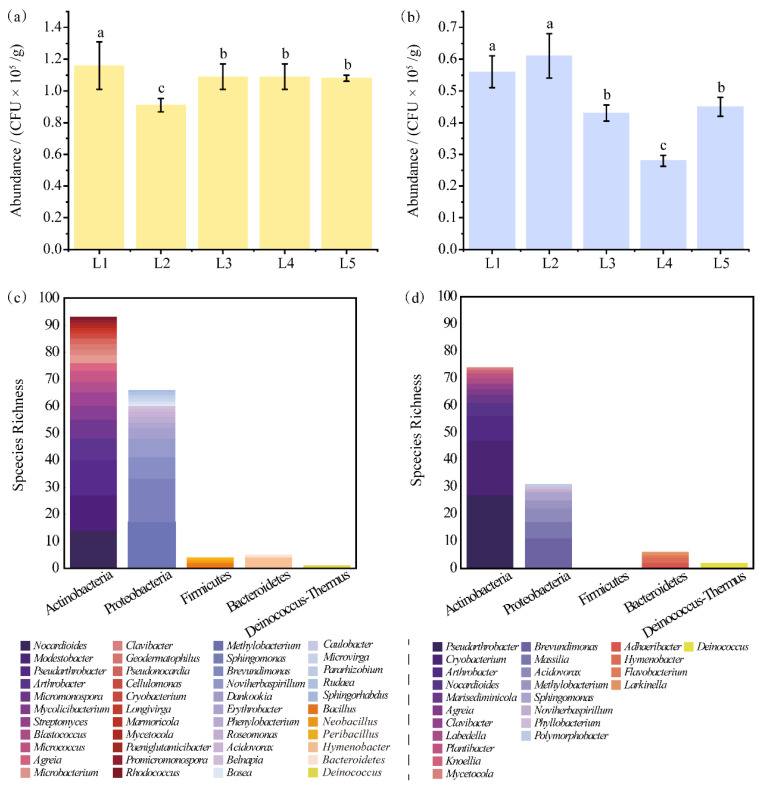
Abundance and species richness of bacteria in the glacier snout of Laohugou Glacier No. 12. Abundance of bacteria at different sites at HT (**a**) and LT (**b**). Species richness of bacteria at HT (**c**) and LT (**d**). Error bars in (**a**,**b**) represent the standard error of three samples collected from each site. Different letters over the bars in (**a**,**b**) indicate significant differences in CFUs at different sites. The Y-axis in (**c**,**d**) represents the number of species.

At the five sampling sites, the average total number of bacteria was 1.07 × 10^5^ CFU/g at 25 °C (HT) and 4.68 × 10^4^ CFU/g at 10 °C (LT). The number of CFUs was significantly different among samples collected from the five sites, and the ranks of CFUs decreased from HT to LT. Sites L1 and L2 had the highest and lowest number of CFUs, respectively, at HT, and had a higher number of CFUs than the other three sites at LT. Site L4 had the lowest number of CFUs at LT (Figure 3a,b).

Based on the morphological evaluation of colonies, 448 isolates were purified from all agar plates with different media at different incubation temperatures. According to the results of 16S rRNA gene sequencing, 259 individual strains were eventually obtained after merging identical isolates from different agar plates. The names, LH*x*M*y* or LL*x*M*y*, of 259 strains were given consecutively. Co-survival was recorded at HT (50%), LT (21.4%), and HT-LT (28.6%). The results of 16S rRNA gene sequencing were deposited in GenBank with the accession numbers OP080742–OP081000. Detailed information on each isolated strain is shown in Table S2.

All isolated strains belonged to five phyla, namely, Actinobacteria, Bacteroidetes, Deinococcus-Thermus, Firmicutes, and Proteobacteria, and 57 genera, including 44 genera belonging to the five phyla at HT and 24 genera belonging to four phyla at LT, except for Firmicutes (Figure 3c,d). 

Actinobacteria and Proteobacteria were the predominant phyla, accounting for 59.2% and 34.3% of the total isolates, respectively. As shown in Figure 3c, Actinobacteria included 22 genera with 93 strains and Proteobacteria had 16 genera with 66 strains at HT. At LT, Actinobacteria included 11 genera with 74 strains and Proteobacteria had 8 genera with 31 strains (Figure 3d). At both HT and LT, Actinobacteria had the highest species richness (55.0% and 65.5%, respectively). The second most abundant phylum was Proteobacteria, accounting for 40.6% and 30.2% of the total isolates at HT and LT, respectively. Bacteroidetes was the third most abundant phylum, accounting for 2.9% of the total isolates (two genera and five strains) at HT and 5.3% of the total isolates (four genera and six strains) at LT. The abundance of Deinococcus-Thermus was lower than that of the other four phyla, with only one genus (*Deinococcus*) with two strains at HT and one strain at LT.

*Pseudarthrobacter* was the most abundant genus, accounting for 14.2% (40) of the total isolates. The genera *Arthrobacter*, *Brevundimonas*, *Cryobacterium*, *Methylobacterium,* and *Sphingomonas* had a similar abundance. However, only one isolate was obtained from each of the 20 genera, including *Belnapia*, *Bosea*, *Caulobacter*, *Cellulomonas*, *Flavobacterium*, *Knoellia*, *Larkinella*, and *Sphingorhabdus* (Figure 3c,d).

Different genera survived at different temperatures. The genera cultured at HT had higher diversity than those cultured at LT and constituted 50% of all genera. Strains of 28 genera were isolated at HT, of which, 12 were represented by one isolate of each genus (Table 1). Only 13 genera were identified at LT, including *Larkinella*, *Aureimonas*, *Marisediminicola*, and *Phyllobacterium* (Table 1). Strains of 16 genera were isolated at both HT and LT. Almost all highly abundant genera were found at both HT and LT, such as *Pseudarthrobacter* with 40 isolates, *Cryobacterium* with 21 isolates, and *Methylobacterium* with 20 isolates (Table 1). Although *Cryobacterium* strains are always found in a cold natural habitat, this study showed that the strains can be cultured at both HT and LT.

### 3.4. Comparison of Bacteria Cultured using Distinct Strategies

Of the four types of media (R2A, TSB, TSB 0.1x, and PYGV), R2A and PYGV were identified as better media for isolating bacteria, irrespective of the culture temperature (Figure S1). At the genus level, approximately 10% and 12.3% of all isolates were cultured in R2A and PYGV media, respectively, at both HT and LT. A total of 10 and 7 unique genera were isolated from samples cultured in R2A and PYGV media at HT, respectively. At LT, 11 genera were isolated from samples cultured in the R2A medium, which were more than those isolated at HT (Figure S1). Detailed information regarding bacterial isolation is mentioned in Table S3. Strains of two unique genera and one unique genus were cultured in a nutrient medium, such as TSB, at HT and LT, respectively (Figure S1). Although TSB was diluted 10 times, it did not serve as an oligotrophic medium. At HT, only *Paeniglutamicibacter* and *Caulobacter* were isolated using TSB, and *Belnapia* and *Pararhizobium* were isolated using TSB 0.1x. At LT, only one unique genus was isolated using TSB or TSB 0.1x (Figures S1 and S3).

**Figure 4:**
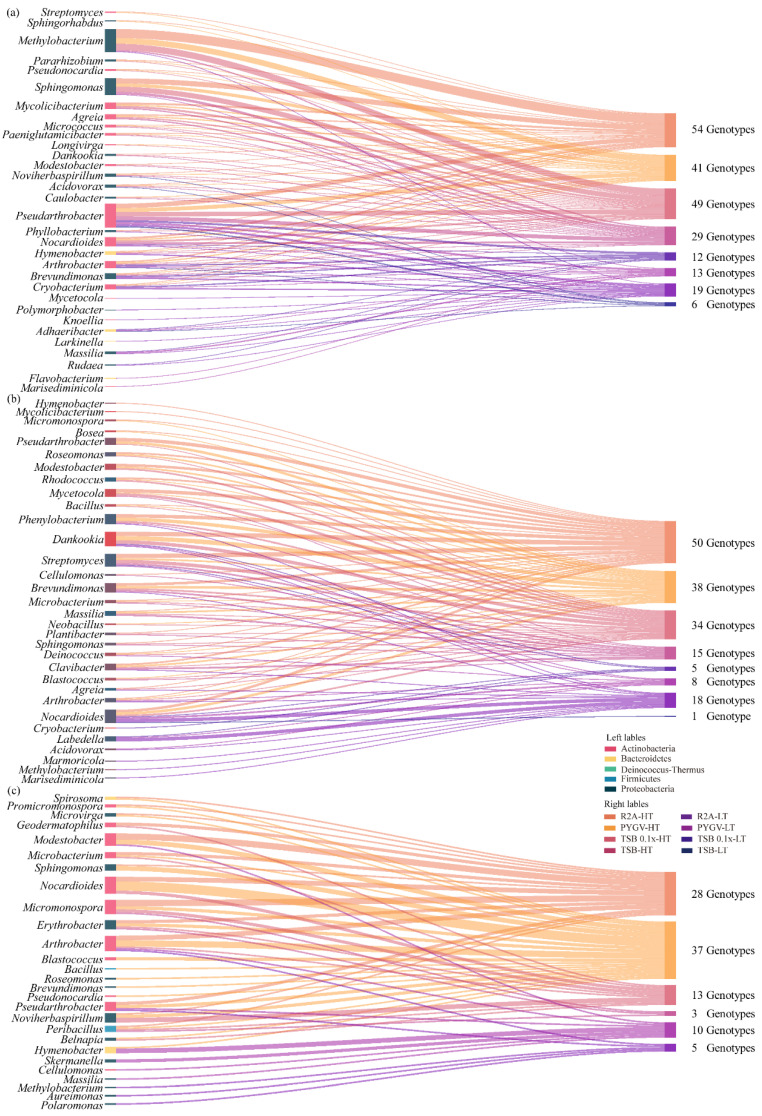
Distribution and diversity of bacteria cultured using different strategies, including different temperatures, media, and pre-treatments. (**a**–**c**) represent the genera of bacteria isolated from normal culture samples (non-pretreatment), samples pre-treated with H_2_O_2_ (oxidation), and samples irradiated with UV-C, respectively.

The diversity of genera isolated at HT was higher than that of genera isolated at LT, irrespective of the treatment (normal culture samples (N), samples pre-treated with H_2_O_2_ (O), or samples irradiated with UV-C (R)) (Figure 4). The number of strains was lower in the R samples than in the N and O samples (Figure 4). Strains of 32 genera were isolated from the N samples, including 7 genera at LT, 11 genera at HT, and 14 genera at both HT and LT (Figure 4a). The total number of genera was the same in the N and O samples; however, the diversity of genera was different at HT and LT (6 genera at LT, 17 genera at HT, and 9 genera at both HT and LT) (Figure 4b). 

**Figure 5:**
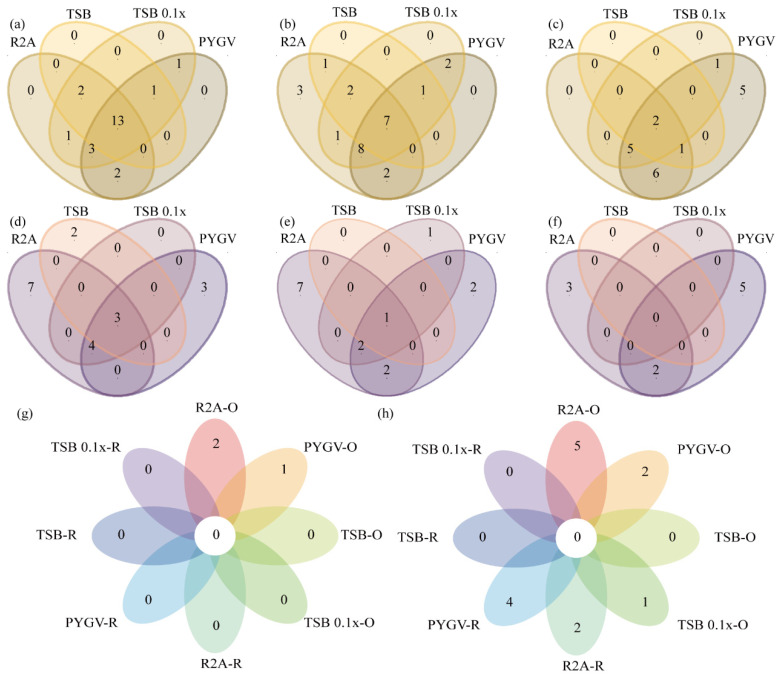
Taxonomic diversity as a function of different culture temperatures, media, and pre-treatments. (**a**–**c**) Venn diagram demonstrating the genera isolated from samples cultured in R2A, PYGV, TSB, and TSB 0.1x media under normal culture (**a**), oxidation (**b**), and radiation (**c**) conditions at HT. (**d**,**e**) Venn diagram demonstrating the genera isolated from samples cultured in R2A, PYGV, TSB, and TSB 0.1x media under normal culture (**d**), oxidation (**e**), and radiation (**f**) conditions at LT. (**g**,**h**) Unique genera isolated from samples cultured in different media under oxidation (O) and radiation (R) conditions at HT (**g**) and LT (**h**).

A total of 27 genera were identified from the R samples, including 6 genera at LT, 17 genera at HT, and 4 genera at both HT and LT (Figure 4c). No strain belonging to any genera was isolated from the R samples incubated with TSB and TSB 0.1x at LT (Figure 4c). Only a few genera were shared among the three types of samples, such as *Marisediminicola* shared between the N and O samples at LT; *Massilia* shared between the N and R samples at LT; *Methylobacterium* shared between the O and R samples at LT; *Modestobacter* and *Paeniglutamicibacter* shared between the N and O samples at HT; *Pseudonocardia* shared between the N and R samples at HT; and *Bacillus*, *Blastococcus*, *Microbacterium*, *Micromonospora*, *Roseomonas,* and *Sphingomonas* shared between the O and R samples at HT (Figure 5a–f and Table S4).

The total abundance of bacteria was higher at HT than at LT in the N samples. More strains were isolated from samples cultured in R2A and TSB 0.1x at HT (24.2% and 22.0%, respectively). *Methylobacterium* was the most abundant genus with 36 isolates in the N samples (35 isolates at HT and 1 isolate at LT) (Figure 4a). The abundance of bacteria in the O and R samples was similar to that in the N samples. The O samples cultured in R2A and TSB 0.1x had a higher diversity of bacteria based on the number of strains (Figure 5b,e). A total of 17 strains belonging to the genus *Dankookia* were isolated from the O samples (Figure 4b). A total of 66 strains belonging to 17 genera were isolated at HT; however, only 12 strains belonging to 6 genera were isolated at LT, including *Labedella* with 6 strains and *Marisediminicola*, *Marmoricola*, *Cryobacterium*, *Acidovorax*, and *Methylobacterium* with 1 strain each (Figure 4). A total of 11 strains belonging to *Nocardioides* were isolated, which was the most abundant genus. Additionally, there were fewer unique taxa (22%) at the genus level in the R samples at HT, including *Cellulomonasm*, *Aureimonas*, *Massilia*, *Methylobacterium,* and *Polaromonas* with one strain each and *Skermanella* with two strains (Figure 4). PYGV was identified as the optimal medium that promoted the isolation of more strains at HT and LT (38.5% and 10.4%, respectively). However, no strain was isolated from the R samples cultured in TSB and TSB 0.1x. 

Venn diagrams were drawn to compare the abundance of bacteria of different genera in the three types of samples at HT and LT (Figure 5g,h). As shown in Figure 5g, only two unique genera, namely, *Mycetocola* and *Pseudonocardia*, were isolated from the O samples cultured in R2A, and one genus, namely, *Sphingorhabdus*, was isolated from the O samples cultured in PYGV at HT. The specific variations in terms of different culture conditions, including distinct media and pre-treatments, indicated that the normal samples at HT had a higher total abundance in the aspect of many genera and the all-culture conditions of LT had more unique taxa than those of HT (Figure 5g,h). The number of unique taxa (14 unique genera) in the N samples was higher at LT than at HT (3 unique genera). However, the number of genera shared by samples cultured in R2A and PYGV was higher at HT than at LT. *Microvirga*, *Modestobacter*, *Promicromonospora*, *Sphingomonas,* and *Spirosoma* were exclusively isolated from samples cultured in R2A and PYGV at HT, except for *Pseudarthrobacter*, which was isolated from samples cultured in R2A and PYGV at LT along with *Arthrobacter* (Figure 5g,h and Table S5). 

### 3.5. High Proportion of Isolated Potential Novel Taxa

The results of 16S rRNA gene sequencing revealed that 34.5% of bacterial isolates had <98.7% similarity with previously reported strains. The abundance of novel species identified at LT (68%) was approximately 2.5 times higher than that of species identified at HT (26.9%) in the N samples (Figure S2a). However, the trend of bacterial abundance was the opposite in the O and R samples, with more bacteria identified at HT than at LT. The number of CFUs of novel species was much higher in samples cultured at HT than in those cultured at LT irrespective of the treatment (Figure S2b). In particular, the proportion of novel species was higher in samples cultured in a medium with different nutrient contents at LT than in those cultured at HT, with the highest proportion of novel species (54%) found in samples cultured in PYGV medium at LT (Figure S2d). The relative proportion of novel species was higher in samples cultured in R2A and PYGV media (46% and 49%, respectively) at HT than in those cultured at LT (36% and 45%, respectively) (Figure S2e). A similar trend was observed after radiation. 

Most closet-type strains of the identified novel species were isolated from normal soil samples, such as landfill, forest, and dry soils (Table S2). Many novel strains (>20%) belonged to the type strains found in extremely cold habitats such as alpine glacier cryoconite, an ice core drilled from the Muztagh glacier, Antarctic soil, some Chinese glaciers, pure ice samples, wildlife on the Qinghai–Tibet plateau and Victoria Upper Glacier, Antarctica, and basal ice. Other strains (34.68%) were closely related to reference-type strains inhabiting extreme environments, such as oil-contaminated soil, ice, extreme hyper-arid Atacama Desert soil, black sand, an approximately 2000-year-old shaft tomb, a marine cyanobacterial mat, and brackish water. Additionally, 10% of the isolated strains belonged to the type strains isolated from plants or plant-associated tissues, such as stem tissues of rice, a decayed elm tree, bean seeds, birch tree, and diseased melon fruits. 

### 3.6. Correlation between the Abundance of Heterotrophic Bacteria and Environmental Factors

**Figure 6:**
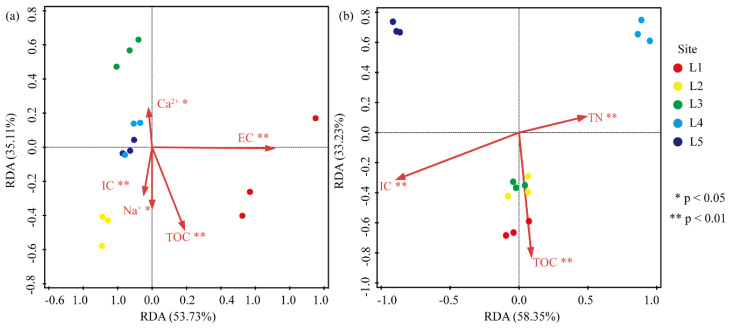
Correlation analyses among the sampling sites, environmental factors, and species richness at HT (**a**) and LT (**b**). EC, electrical conductivity; TN, total nitrogen; IC, inorganic carbon; TOC, total organic carbon.

RDA was performed to examine the correlation between environmental factors and the abundance of bacteria isolated from soil samples collected from all sites. The results revealed that the content of TOC and IC had a highly significant impact (*p* < 0.01) on CFUs in samples cultured at both HT and LT (Figure 6). Moreover, the concentration of Na^+^ and Ca^2+^ ions had a significant impact (*p* < 0.05) on CFUs, and EC had a highly significant impact (*p* < 0.01) on CFUs in samples cultured at HT (Figure 6a). Inorganic ions did not show a significant correlation with CFUs at LT. In addition to TOC and IC, TN showed a highly significant correlation (*p* < 0.01) with CFUs in samples cultured at LT (Figure 6b). 

The Spearman correlation analysis showed that environmental factors were significantly correlated with the abundance of some phylotypes. Among the analyzed strains, 436 stains were significantly positively correlated, and 371 strains were significantly negatively correlated with one or several environmental factors (Figure S3). The number of significant positive correlations between bacterial abundance and the concentration of TOC, TN, pH, Ca^2+^ ions, and Mg^2+^ ions was higher than the number of significant negative correlations. The abundance of Proteobacteria was significantly positively correlated with the content of TOC and TN (50.5% and 51.8%, respectively) (Figure S3). 

The concentration of IC, EC, WC, and K^+^ ions showed more significant negative correlations than positive correlations. In particular, no significant positive correlation was observed between the concentration of K^+^ ions and bacterial abundance; however, 43 significant negative correlations were observed. Approximately 76.7% of significant negative correlations were observed between the abundance of Actinobacteria and the concentration of K^+^ ions (Figure S3).

### 3.7. Growth Temperature Range of Each Isolated Heterotrophic Bacterium

**Figure 7:**
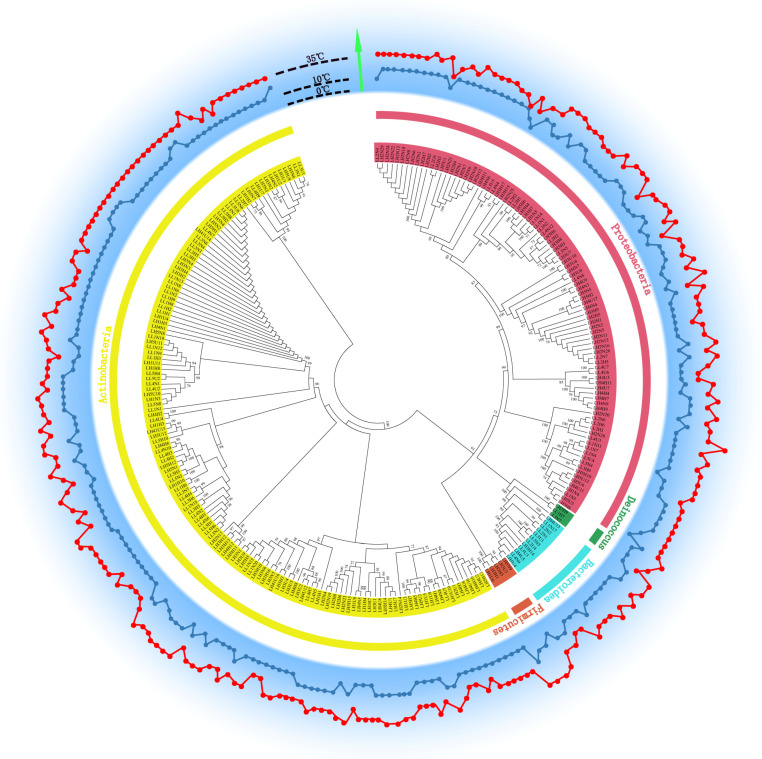
Growth temperature range of each isolated strain. The innermost circle is a neighbor-joining phylogenetic tree based on 16S rRNA gene sequencing of all isolated strains. Numbers on the tree represent the proportion of bootstrap samples derived from 1000 replications and show bootstrap values higher than 70%. Bar, <0.01 substitutions per nucleotide position. The red and blue dots represent the highest and lowest growth temperatures, respectively, in the outer circle.

A temperature range (5, 10, 20, 25, 30, 35, 40, 45, and 50 °C) was selected to assess the tolerated growth temperature range of each bacterial strain. As shown in Figure 7, all strains could grow above 5 °C, and the optimal temperature for their growth was higher than 10 °C and, occasionally, 20 °C (68.7% of strains) (Table S2). Only 16 strains (3 Proteobacteria and 13 Actinobacteria strains) could grow at 5 °C, and all of them were isolated from samples cultured at LT. The highest growth temperature was 50 °C for three strains of Firmicutes at HT.

### 3.8. Radiation Resistance of Isolated Heterotrophic Bacteria

**Figure 8:**
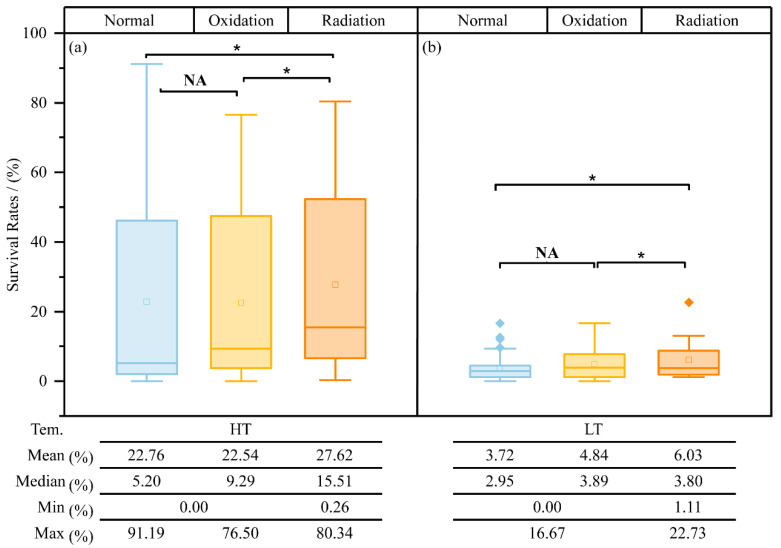
Assessment of resistance to 100-J/m^2^ UV-C radiation in strains isolated from samples with different pre-treatments at HT (**a**) and LT (**b**). NA, no significant correlation; asterisks (*), significant difference (*p* < 0.05).

The study region receives substantial irradiation throughout the year. Therefore, we tested the ability to resist radiation in strains isolated from samples with different pre-treatments (the N, O, and R samples). After irradiation at 100 J/m^2^, the ability to resist radiation was stronger in strains isolated at HT than in those isolated at LT. Although the strains isolated from the N samples also had a strong ability to resist radiation, the strains isolated at HT after irradiation had a higher mean survival rate (27.62%). As shown in Figure 8, the same trend was observed in strains isolated at HT after irradiation, and the highest survival rate was 22.73%. Moreover, the survival rates of strains isolated from the N and O samples were significantly lower than those of strains isolated from the R samples (*p* < 0.05) at both HT and LT. 

## 4. Discussion

Glacial habitats are considered huge treasure troves of microbial ecology. Owing to the retreat of glaciers, glacier snouts are exposed as a completely bare new terrain with variable environmental conditions. Bacterial communities in this new terrain formed by primary succession are quite unstable, and microorganisms inhabiting the original glacier snout are stress-tolerant “opportunists” with selective growth [4,36]. Such microorganisms can grow under fluctuating environmental conditions. This study provided insights into bacterial diversity and the temporary survival of “opportunists” driven by various environmental factors during primary succession at the glacial snout of Laohugou Glacier No. 12. 

### 4.1. Comparison of Bacteria Isolated from the Moraine of Laohugou Glacier No. 12 and Other Glacial Niches

The diverse bacteria isolated from the glacial snout of Laohugou Glacier No. 12 on the northern slope of the western Qilian Mountains were similar to those reported in previous studies [6,12]. Actinobacteria and Proteobacteria were the predominant phyla both at HT and LT. More than 50% of bacterial isolates belonged to Actinobacteria (Figure 3c,d). As the oldest living organisms, Actinobacteria can survive at extreme temperatures [37,38] and adapt to and predominate a cold oligotrophic environment by forming the mycelia required for acquiring nutrients [39]. For survival, Actinobacteria can actively decompose organic materials in cold oligotrophic soil [40], materials from dead microbes, arthropods, fungal spores, pollen, and other organic substances deposited by air or glacial meltwater on the glacial snout [41,42]. A high abundance of Actinobacteria has been reported in other glacial ecosystems, such as Mount Everest glacial meltwater in the Tibet Autonomous Region [21], retreat soil and meltwater of Baishui glacier No. 1 located in Yulong Snow Mountain [11], the Chongce ice cap of the west Kunlun Mountains [16,43], the Hailuogou Glacier retreat near the Minya Konka [36], and the Damma glacier receding forefield located in the Central Alps [5], indicating their ubiquitous presence in diverse glacial ecosystems. Proteobacteria was also the pioneer bacterial taxa dominating the recent stage. Proteobacteria strains have been reported to be abundant in deglaciated soil in the Peruvian Andes and Himalayas [14,44] and the subglacial sediments from an Alaskan glacier [45]. Yergeau et al. suggested that the rapid growth of Proteobacteria is attributed to its high abundance in glacier ecosystems [46]. However, Ali et al. reported that the diversity of Firmicutes (58.33%) was higher than that of Actinobacteria (23.0%) and Proteobacteria (14.6%) in the same study region and similar sampling sites [17]. Different culture conditions, such as the culture temperature, the nutritional composition of the culture, and variations in environmental factors can influence the results.

In this study, the species richness of Firmicutes was 1.28 × 10^3^ CFUs/g, accounting for approximately 1.2% of all isolates at HT. However, no strains were isolated at LT (Figure 3b). Previous studies on other glaciers have reported that the abundance of Firmicutes is higher than that of Actinobacteria and Proteobacteria in the newly exposed moraine of a glacier terminus [6,9,14,21,47]. However, a lower abundance does not mean the absence of bacteria. The abundance of Firmicutes is higher than that of Actinobacteria and Proteobacteria in the basal ice of the Qinghai–Tibet Plateau [3]. Differences between ecological niches may significantly contribute to differences among bacterial communities [6,21]. The higher abundance of Firmicutes [26,48,49] can be attributed to the endospore-forming ability of several strains of Firmicutes, which helps to resist potentially harsh conditions [50]. Recently, Sajjad et al. [11] reported that Firmicutes can continuously migrate from newly exposed moraines to early retreat regions via glacial meltwater or melting ice and cannot recolonize in the moraines owing to selective migration toward meltwater [17]. Bhattacharya et al. also indicated that the shorter the time to melt at the toe of the glacier, the higher the dominance of Actinobacteria and Proteobacteria [10]. Therefore, the mobile nature of meltwater may be one of the key reasons for spatial variations in bacterial communities, especially in newly exposed moraines [21].

The competitive interaction among bacterial communities under nutrient-limited conditions determines the dominance of specific taxa [6]. Spatial gradients in environmental factors are the main drivers of bacterial diversity and community composition [9]. A majority of Proteobacteria and Actinobacteria strains are copiotrophic with higher growth rates, a greater degree of variability in population size, and lower substrate affinities [51]. Actinobacteria and Proteobacteria can actively participate in carbon assimilation and mineralization pathways [3]. In this study, the taxonomic analysis showed that *Methylobacterium* belonging to Actinobacteria and *Pseudarthrobacter* belonging to Proteobacteria phyla were the predominant genera at HT and LT, respectively (Figure 3c,d). *Methylobacterium* and *Pseudarthrobacter* species can fix nitrogen through denitrification [52]. Enhanced nitrogen fixation may lead to nitrogen accumulation in a cold ecosystem, which promotes microbial growth at a later stage [22]. Therefore, *Methylobacterium* and *Pseudarthrobacter* species can metabolize carbon and nitrogen sources in moraines for succession, especially in newly exposed moraines with higher TC and TN content than the earlier regressive region [6]. RDA showed that TOC and TN had a significant positive correlation with the abundance of bacteria, irrespective of the culture temperature (Figure 6). In newly-exposed moraines at glacier snouts, although local soil and environmental factors are the driving indicators that define bacterial diversity and community structure [9], fluctuations in temperature, nutrient concentrations, oxidation, and irradiation during glacier retreat lead to more instability at the lower level of systematic taxonomy, and bacteria may respond to such stresses [4]. 

### 4.2. Possible Effects of Temperature Changes on Bacteria Inhabiting the Newly Exposed Moraine 

Compared with snow or ice, which is a relatively constant glacial habitat for cold-adapted microbial biota, newly melting glacial ice is highly dynamic in terms of ecosystem development and faces primary succession, especially at glacier snouts [15,16]. With melting at glacier snouts, the previously extreme cold (usually <0 °C) and anaerobic or microaerobic subglacial ecological environment is gradually exposed [13,19]. Most bacteria in oligotrophic and extremely low-temperature habitats are sensitive to fluctuations in temperature and may become dormant or die [13]. Fewer bacteria can survive in newly melting ice than in early retreating habitats [3,6]. In this study, although several microorganisms could grow at 10 °C (LT), a higher number of microorganisms grew at 25 °C (HT). At the genus level, 50% of all isolates grew only at HT, 21.4% grew only at LT, and 38.6% grew at both LT and HT. This phenomenon seems less plausible in an extremely cold habitat with temperatures of <10 °C (Figure 3a,b), especially in terms of the number of culturable taxa. Fluctuations in temperature may limit the growth of indigenous bacteria and lead to their death. As a result, only a few bacteria that can rapidly adapt to such changes may grow well or gradually [16,19]. The active metabolism of cells may help to adapt to sharply increasing temperatures in subglacial permafrost [53], which is in contrast to the slow metabolism of bacteria in extremely cold ecological environments [54]. Extremely slow growth at low temperatures and weaker metabolism may be one of the survival features adopted by bacteria residing in cold glaciers [21]. Moreover, the cells get warm and rehydrate owing to increasing temperatures, thus helping bacteria to adapt to advanced metabolism for growth [53,54]. Therefore, such bacteria can be cultured at both 10 °C and 25 °C (much higher than the average temperature). These bacteria can rapidly respond to the coercion for adaptation; however, non-responsive bacteria are also present in a big “seeds pool” [4]. The initially non-responsive bacteria may gradually respond to such temperature alterations, fail to adapt to increasing temperatures, and even not be colonized here [4,5,6,19]. 

In this study, a majority of bacteria isolated from the newly exposed moraine at the glacier snout belonged to a nonglacial environment (Table S2). Only approximately 10% of the isolates belonged to glacial ecological environments; however, >50% of their nearest neighbors belonged to adverse or stressed habitats, such as oil-contaminated soil, rocks, and black sand (Table S2). Glacial meltwater may be the main source of bacteria in newly exposed moraines [11]. Other sources may include plant, eolian deposition, precipitation events, glacial dust, and aerosol [4,5,21]. In this study, the nearest neighbors of some strains belonged to extreme hyper-arid Atacama Desert soil and black sand [55,56]. Various sources of bacteria may be another reason for the presence of a large number of bacteria in samples cultured at 10 °C and 25 °C. A few “opportunists” can be cultured at <10 °C, all isolated strains can be cultured at 20 °C, and >90% of all isolates can be cultured at >30 °C (Figure 7). 

### 4.3. Temporary Survival and Potential Roles of Bacteria in Response to Different Environmental Stresses

Previous studies have reported that no single species can tolerate both the disruption associated with the disturbance of original ecological niches and the ensuing competition of recolonization [57,58]. We hypothesized that bacteria in various ecological niches respond differently to environmental stress. According to a well-known ecological theory, everything is everywhere, but the environment selects [59]. Highly fluctuating environmental conditions significantly drive the highly dynamic variations in bacteria [19].

In addition to temperature, nutrient availability impacts the bacterial community [4,60,61]. This study showed that oligotrophic media (R2A and PYGV) rejuvenated more bacteria than eutrophic media (TSB and TSB 0.1x), and the genera isolated from samples cultured in different media had high diversity, irrespective of the culture temperature (Figure 5a–f). However, the nutrient composition of the oligotrophic media used in this study is richer than that of the background region, which corresponds to increased nutrient stress in the process of isolating more bacteria. Therefore, the isolated bacteria were better adapted to withstand nutrient depletion in the culture media [4]; however, some may prefer to live in their original ecological niches. Surviving in high-nutrition environments can be one of the survival traits because the nutritional composition of the glacier snout after melting may be richer than that before deglaciation [10]. In addition, the stable resident bacteria make glacial retreats an important carbon sink [6]. The “opportunists” promote bacterial diversity at the glacier snout; as a result, the survival of bacteria is greatly increased. These opportunists can decompose organic carbon (OC) stored in glaciers into greenhouse gases such as CO_2_ and CH_4_, which enhances the transformation of the newly melting ice from a carbon sink into a carbon source [3,6,13]. As primary producers, whether bacterial survivors could be the extra source of the net CO_2_ or sinks needed to be explored further [13,62,63].

In addition to the basic variations, oxidative stress and direct UV irradiation constrain the growth of bacteria living in this region [13,64,65]. In this study, the number of identified genera was the same in the N and O samples (Table S4); however, significant differences were observed in diversity and species abundance (Table S4). Owing to the exposure of oxygen, the old OC nourished selective growth aerobic bacteria which gathered on the newly melted soil by decomposing and using OC [6,21]. The higher content of organic matter (OM) in the newly melted ice than in the sandy soil in the nearby river also indirectly verified this phenomenon [6]. As shown in Figure 8, the abundance of bacteria was lower in the R samples than in the N and O samples (Figure 8). Martin et al. suggested that strong UV radiation can reduce the activity of bacteria in Antarctic Sea ice and may lead to bacterial dormancy or death [66]. The ecological niches of most radiation-resistant bacteria are directly exposed to radiation [34,38,67,68]. This study showed that most strains isolated from the R samples at HT were strongly resistant to radiation (Figure 8). Bacteria residing in ice at glacier snouts can produce photoprotective molecules to resist UV radiation [69]. Moreover, radiation stress can induce selective growth in potential radiation-resistant bacteria; these bacteria can absorb energy produced by UV radiation and captured by the chromophores of photoactive proteins [70]. A high proportion of isolates (86%) can form pigmented colonies to protect themselves against solar irradiation [71,72,73,74]. Vitousek and White suggested that pioneer organisms should often possess heightened tolerance to extreme environmental conditions [75]. Although tolerating environmental stresses for selective growth involves potentially high energy costs [76], such survival traits can increase the ability of bacteria to recolonize in regions to which they are not adapted, which can be explained as follows: “the higher the environmental stress, the more the survival choices and the higher the diversity”.

## 5. Conclusions

The glacier snout of Laohugou Glacier No. 12 has a highly diverse bacterial community. A total of 259 bacterial strains were isolated from soil samples collected from five sites around the glacier snout along the altitude of 4273–4300 m a.s.l. These strains belonged to 5 phyla and 57 genera. Actinobacteria was the most abundant phylum, followed by Proteobacteria, at both HT and LT. Deinococcus-Thermus had the lowest abundance at HT, and Firmicutes strains were not isolated at LT. To the best of our knowledge, this study is the first to report on the ecological traits of bacterial communities within non-glacial niches and their traits of temporary survival and response to survival stress in a newly exposed moraine with variable environmental conditions. The isolated bacterial strains belonged to various original ecological niches beyond the glacier. Although these bacteria survived temporarily, they did not adapt to the new environment. The higher abundance and diversity of bacteria in samples cultured using different strategies indicated that high fluctuations in environmental factors constrain the survival of bacteria; however, some rapidly adapting “opportunists” may respond to such stress for growth. The presence of such “opportunists” indicates the instability of early succession, and they have high metabolic activity at the glacier snout. These opportunists can accelerate the transformation of the newly exposed moraine from a carbon sink to a carbon source during glacier retreat, thus improving bacterial adaptation and diversity in response to later succession periods. Overall, this study provides a theoretical basis for the study of microorganisms surviving in the transition region between glaciers and moraines to improve the understanding of processes related to the carbon cycle in the context of global warming. 

## Figures and Tables

**Table 1:** Taxonomic diversity at different culture temperatures used. Venn diagram comparing the genera isolated at HT and LT (Left figure).

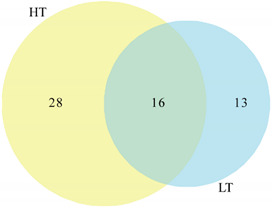	HT	*Blastococcus* *Geodermatophilus* *Marmoricola* *Microbacterium* *Micrococcus* *Micromonospora* *Mycolicibacterium*	*Promicromonospora* *Pseudonocardia* *Rhodococcus* *Streptomyces* *Phenylobacterium* *Paeniglutamicibacter* *Sphingorhabdus*	*Caulobacter* *Dankookia* *Erythrobacter* *Microvirga* *Pararhizobium* *Roseomonas* *Longivirga*	*Bacillus* *Neobacillus* *Peribacillus* *Belnapia* *Rudaea* *Bosea* *Spirosoma*
	Shared	*Mycetocola* *Pseudarthrobacter* *Hymenobacter* *Modestobacter*	*Methylobacterium* *Noviherbaspirillum* *Brevundimonas* *Cellulomonas*	*Deinococcus* *Acidovorax* *Sphingomonas* Arthrobacter	*Cryobacterium* *Clavibacter* *Agria* *Nocardioides*
	LT	*Larkinella* *Aureimonas* *Marisediminicola*	*Phyllobacterium* *Polymorphobacter* *Flavobacterium*	*Skermanella* *Polaromonas* *Knoellia*	*Massilia* *Plantibacter* *Adhaeribacter*

## Data Availability

The datasets generated for this study can be found in GenBank under the accession numbers OP080742–OP081000.

## Supplementary Materials

The following are available online at https://www.mdpi.com/article/10.3390/biology11111555/s1, Figure S1. Taxonomic diversity as a function of the culture media used. Venn diagram comparing the genera isolated in R2A, PYGV, TSB, and TSB 0.1x media at HT (**a**) and LT (**b**). Figure S2. The percentage (**a**) and abundance (**b**) of the culturable new species at HT and LT. Frequency of total possible new species and known species comparing the genera isolated in R2A, PYGV, TSB, and TSB 0.1x media at HT and LT (**c**), (**d**–**f**) were the results of different pretreatments with normal, oxidation, and radiation, respectively, under the same conditions as (**c**). Figure S3. Co-occurrence networks of culturable bacterial species associated with environmental factors. Each edge means significant correlations between the nodes. Within the edges, the solid lines and the dashed lines represent positive and negative correlations, respectively, while the blue and red lines refer to Spearman’s correlation at the levels of *p* < 0.05 and *p* < 0.01, respectively. Table S1. Media was used for isolation in this study. Table S2. Taxonomic affiliations of strains, determined by phylogenetic analysis of 16S rRNA. Table S3. List of genera represented in the Venn diagram of Figure S1. Table S4. List of genera represented in the Venn diagram of Figure 5a,f. Table S5. List of genera represented in the Venn diagram of Figure 5g,h.
