# Supplementary material for: Temporary Survival Increasing the Diversity of Culturable Heterotrophic Bacteria in the Newly Exposed Moraine at a Glacier Snout
Source: Biology (Basel). 2022 Oct 24;11(11):1555. doi: 10.3390/biology11111555 (PMC9687651; doi:10.3390/biology11111555)
Supplement: Supplementary file 1 [file biology-11-01555-s001.zip › biology-1878978-supplementary.pdf]

Supplementary Materials

Figures: Figure S1-Figure S3

Tables: Table S1-Table S5

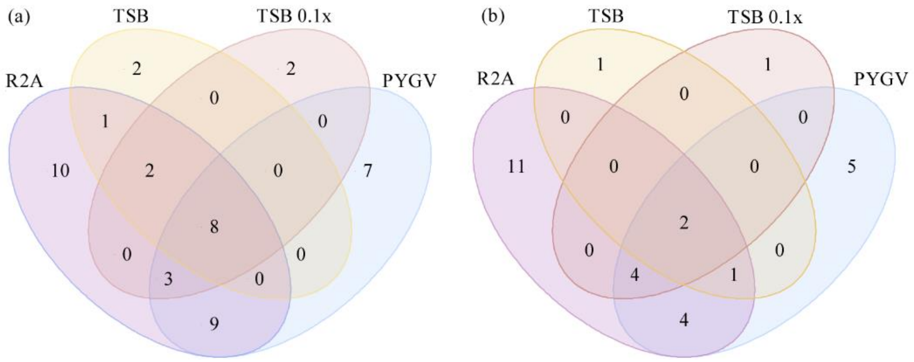

Figure S1. Taxonomic diversity as a function of the culture media used. Venn diagram comparing the genera isolated in R2A, PYGV, TSB, and TSB 0.1x media at HT (a) and LT (b).

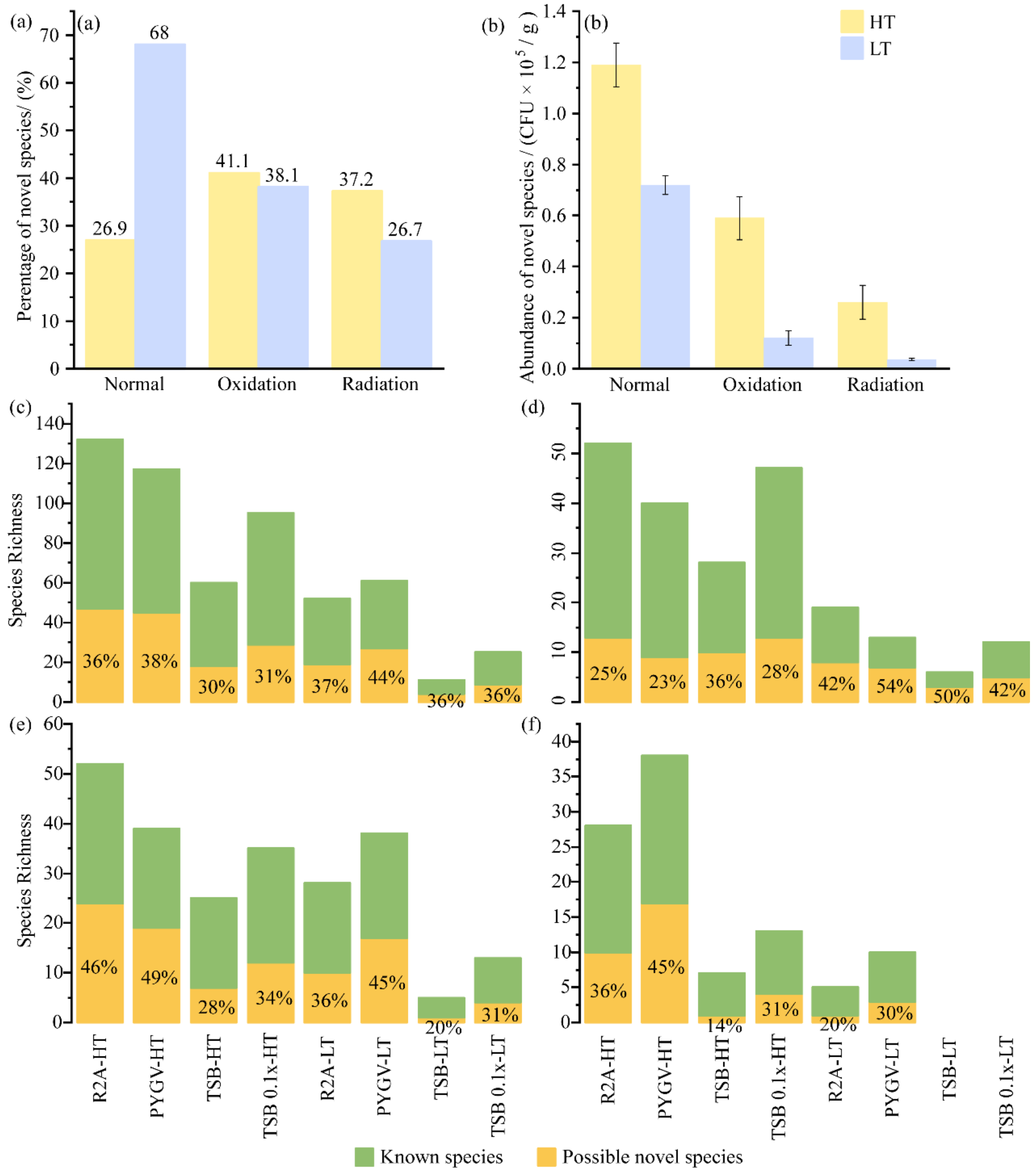

Figure S2. The percentage (a) and abundance (b) of the culturable new species at HT and LT. Frequency of total possible new species and known species comparing the genera isolated in R2A, PYGV, TSB, and TSB 0.1x media at HT and LT (c), (d) – (f) were the results of different pretreatments with normal, oxidation and radiation respectively under the same conditions as (c).

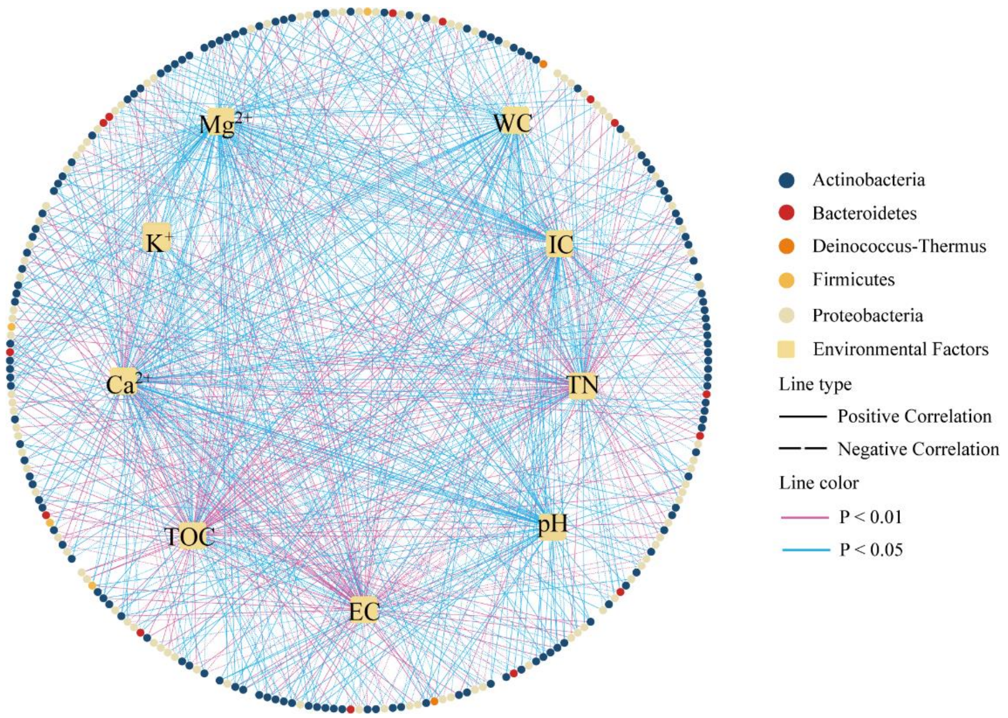

Figure S3. Co-occurrence networks of culturable bacterial species associated with environmental factors. Each edge means significant correlations between the nodes. Within the edges, the solid line and the dashed lines represent positive and negative correlations, respectively, while the blue and red lines refer to Spearman's correlation at the levels of $p < 0.05$ and $p < 0.01$, respectively.

Tables Table S1-Table S5

Table S1. Media was used for isolation in this study

Media	Composition
**R2A (Reasoner's 2A agar)**	Yeast extract 0.5 g, peptone 0.5 g, casamino acid 0.5 g, glucose 0.5 g, soluble starch 0.5 g, K_2HPO_4 0.3 g, $MgSO_4 \cdot 7H_2O$ 0.05 g, Na-pyruvate 0.3 g, distilled water 1000 ml, agar 15 g, pH 7.2 ± 0.2.
**PYGV**	Mineral salt solution. ("Hutner/Cohen-Bazire") 20 ml, peptone 0.25 g, yeast extract 0.25 g, agar 15 g, distilled water 965 ml; sterilize 20 min./121 °C. After cooling to 60 °C add to the medium: glucose sol. (2.5%, sterile-filtered) 10ml, vitamin solution. (Double conc.) 5ml.<br>Adjust pH to 7.5 (the medium is only weakly buffered; one needs approx. 10 drops/L medium of 6 N KOH).<br>**Components of Mineral salt solution:**<br>Nitrilotriacetic acid 10 g, $MgSO_4 \cdot 7H_2O$ 29.7 g, $CaCl_2 \cdot 2H_2$ O 3.34g, $Na_2MoO_4 \cdot 2H_2O$ 12.67 mg, $FeSO_4 \cdot 7H_2O$ 99 mg, Metal salt sol. "Metals 44" 50 ml, distilled water 900ml.<br>**Components of Metals 44:**<br>Na-EDTA 250 mg, $ZnSO_4 \cdot 7H_2O$ 1095 mg, $FeSO_4 \cdot 7H_2O$ 500 mg, $MnSO_4 \cdot H_2O$ 154 mg, $CuSO_4 \cdot 5H_2O$ 39.2 mg, Co $(NO_3)_2 \cdot 6H_2O$ 24.8 mg, $Na_2B_4O_7 \cdot 10H_2O$ 17.7 mg, distilled water 1000ml.<br>Dissolve the EDTA and add a few drops of concentrated H_2SO_4 to retard precipitation of the heavy metal ions.<br>**Components of vitamin solution (double conc.):**<br>Biotin 4 mg, folic acid 4 mg, pyridoxine-HCl 20 mg, riboflavine 10 mg, thiamine-$HCl \cdot 2H_2O$ 10 mg, nicotinamide 10 mg, D-Ca-pantothenate 10mg, vitamin B_{12} 0.2 mg, p-Aminobenzoic acid 10 mg, distilled water 1000 ml.
**TSA (tryptic soy agar)**	Tryptone 17 g, soytone 3 g, NaCl 5 g, glucose 2.5 g, K_2HPO_4 0.5 g, distilled water 1000 ml, agar 15 g, pH 7.5 ± 0.2.
**TSA 0.1x (tryptic soy agar diluted ten times)**	Tryptone 1.7 g, soytone 0.3 g, NaCl 0.5 g, Glucose 0.25 g, K_2HPO_4 0.05 g, distilled water 1000 ml, agar 15 g, pH 7.5 ± 0.2.

Table S2 Taxonomic affiliations of strains, determined by phylogenetic analysis of 16S rRNA

Strains	No. of isolates	Genbank accession no.	Phylogenetically nearest taxon based on 16S rRNA	Phylum	Identity %	Sources of the nearest taxon
**LH1H1**	4	OP080742	*Marmoricola silvestris*	Actinobacteria	97.6	lpine forest soil.
**LH1H2**	3	OP080743	*Clavibacter michiganensis subsp. phaseoli*	Actinobacteria	99.7	bean seeds (Phaseolus vulgaris L.)
**LH1H3**	2	OP080744	*Microbacterium hatanonis*	Actinobacteria	98.5	contaminant of hairspray
**LH1H4**	2	OP080745	*Micromonospora equina*	Actinobacteria	97.9	soil
**LH1H5**	3	OP080746	*Pseudarthrobacter psychrotolerans*	Actinobacteria	99.1	Antarctic soil
**LH1H7**	3	OP080747	*Micromonospora yangpuensis*	Actinobacteria	98.0	a cup-shaped sponge collected at Dachan reef
**LH1H8**	3	OP080748	*Arthrobacter zhaoguopingii*	Actinobacteria	98.0	plateau wildlife on the Qinghai-Tibet Plateau of PR China
**LH1H10**	4	OP080749	*Clavibacter michiganensis subsp. chilensis*	Actinobacteria	99.7	bean seeds (Phaseolus vulgaris L.)
**LH1N3**	4	OP080750	*Arthrobacter ruber*	Actinobacteria	99.4	glacier ice
**LH1N4**	4	OP080751	*Noviherbaspirillum psychrotolerans*	Proteobacteria	98.2	oil-contaminated soil
**LH1N5**	4	OP080752	*Brevundimonas alba*	Proteobacteria	98.2	oligotrophic environments
**LH1U1**	2	OP080753	*Arthrobacter luteolus*	Actinobacteria	99.3	contaminant soil
**LH1U2**	1	OP080754	*Arthrobacter zhaoguopingii*	Actinobacteria	99.3	plateau wildlife on the Qinghai-Tibet Plateau of PR China
**LH1U3**	1	OP080755	*Blastococcus litoris*	Actinobacteria	97.7	soil
**LH1U4**	1	OP080756	*Pseudarthrobacter psychrotolerans*	Actinobacteria	99.1	Antarctic soil
**LH1U5**	2	OP080757	*Geodermatophilus normandii*	Actinobacteria	99.4	air
**LH1U6**	1	OP080758	*Bacillus tequilensis*	Firmicutes	98.8	air
**LH1U7**	2	OP080759	*Promicromonospora iranensis*	Actinobacteria	99.5	soil
**LH1U8**	0	OP080760	*Pseudonocardia hydrocarbonoxydans*	Actinobacteria	97.6	soil
**LH1U9**	3	OP080761	*Geodermatophilus obscurus*	Actinobacteria	99.0	plateau wildlife on the Qinghai-Tibet Plateau of PR China
**LH1U10**	2	OP080762	*Modestobacter caceresii*	Actinobacteria	98.5	stem tissues of rice,
**LH1U11**	3	OP080763	*Arthrobacter zhaoguopingii*	Actinobacteria	98.8	plateau wildlife on the Qinghai-Tibet Plateau of PR China
**LH2H1**	3	OP080764	*Sphingomonas melonis*	Proteobacteria	99.3	yellow Spanish melon fruits
**LH2H2**	3	OP080765	*Methylobacterium oryzae*	Proteobacteria	98.8	stem tissues of rice,
**LH2H3**	3	OP080766	*Methylobacterium jeotgali*	Proteobacteria	99.5	jeotgal, a traditional Korean fermented seafood
**LH2H4**	3	OP080767	*Methylobacterium aquaticum*	Proteobacteria	99.3	almost any freshwater environment where dissolved oxygen exists
**LH2H5**	3	OP080768	*Sphingomonas melonis*	Proteobacteria	98.6	yellow Spanish melon fruits
**LH2H6**	3	OP080769	*Mycolicibacterium sphagni*	Actinobacteria	97.4	pollutant envirments
**LH2H7**	2	OP080770	*Methylobacterium oryzae*	Proteobacteria	98.8	stem tissues of rice,

Strains	No. of isolates	Genbank accession no.	Phylogenetically nearest taxon based on 16S rRNA	Phylum	Identity %	Sources of the nearest taxon
**LH2H8**	2	OP080771	*Mycolicibacterium sphagni*	Actinobacteria	97.5	pollutant envirments
**LH2N1**	2	OP080772	*Methylobacterium aquaticum*	Proteobacteria	99.2	almost any freshwater environment where dissolved oxygen exists
**LH2N2**	4	OP080773	*Methylobacterium oryzae*	Proteobacteria	99.0	stem tissues of rice,
**LH2N3**	3	OP080774	*Sphingomonas melonis*	Proteobacteria	98.9	yellow Spanish melon fruits
**LH2N4**	4	OP080775	*Mycolicibacterium sphagni*	Actinobacteria	97.9	pollutant envirments
**LH2N5**	3	OP080776	*Sphingomonas melonis*	Proteobacteria	98.7	yellow Spanish melon fruits
**LH2N6**	3	OP080777	*Methylobacterium oryzae*	Proteobacteria	99.4	stem tissues of rice,
**LH2N7**	3	OP080778	*Methylobacterium aquaticum*	Proteobacteria	99.5	almost any freshwater environment where dissolved oxygen exists
**LH2N8**	3	OP080779	*Methylobacterium oryzae*	Proteobacteria	98.8	stem tissues of rice,
**LH2N9**	2	OP080780	*Methylobacterium aquaticum*	Proteobacteria	99.3	almost any freshwater environment where dissolved oxygen exists
**LH2N10**	3	OP080781	*Methylobacterium oryzae*	Proteobacteria	99.5	stem tissues of rice,
**LH2N11**	3	OP080782	*Sphingomonas melonis*	Proteobacteria	98.8	yellow Spanish melon fruits
**LH2N12**	2	OP080783	*Methylobacterium aquaticum*	Proteobacteria	98.9	almost any freshwater environment where dissolved oxygen exists
**LH2N13**	2	OP080784	*Methylobacterium oryzae*	Proteobacteria	99.3	stem tissues of rice,
**LH2N14**	2	OP080785	*Longivirga aurantiaca*	Actinobacteria	97.8	lake sediment
**LH2N15**	2	OP080786	*Sphingomonas melonis*	Proteobacteria	99.0	yellow Spanish melon fruits
**LH2N16**	4	OP080787	*Sphingomonas melonis*	Proteobacteria	98.5	yellow Spanish melon fruits
**LH2N17**	2	OP080788	*Streptomyces deserti*	Actinobacteria	98.7	desert soil
**LH2N18**	3	OP080789	*Methylobacterium aquaticum*	Proteobacteria	99.1	almost any freshwater environment where dissolved oxygen exists
**LH2N19**	2	OP080790	*Mycolicibacterium sphagni*	Actinobacteria	97.4	pollutant envirments
**LH2N20**	2	OP080791	*Rudaea cellulosilytica*	Proteobacteria	98.5	soil
**LH2N21**	3	OP080792	*Methylobacterium aquaticum*	Proteobacteria	99.4	almost any freshwater environment where dissolved oxygen exists
**LH2N22**	2	OP080793	*Methylobacterium oryzae*	Proteobacteria	99.0	stem tissues of rice,
**LH2N23**	3	OP080794	*Mycolicibacterium arabiense*	Actinobacteria	99.1	Lake Dianchi, Yunnan Province, China
**LH2N24**	2	OP080795	*Methylobacterium oryzae*	Proteobacteria	98.8	stem tissues of rice,
**LH2N25**	3	OP080796	*Caulobacter vibrioides*	Proteobacteria	99.0	mountain
**LH2N26**	2	OP080797	*Sphingomonas melonis*	Proteobacteria	99.0	yellow Spanish melon fruits
**LH2N28**	4	OP080798	*Acidovorax soli*	Proteobacteria	98.9	landfill soil
**LH2N29**	1	OP080799	*Methylobacterium oryzae*	Proteobacteria	99.3	stem tissues of rice,
**LH2U1**	2	OP080800	*Modestobacter caceresii*	Actinobacteria	98.8	stem tissues of rice,
**LH3H1**	3	OP080801	*Bacillus velezensis*	Firmicutes	99.4	plant
**LH3H2**	3	OP080802	*Pseudarthrobacter psychrotolerans*	Actinobacteria	99.4	Antarctic soil
**LH3H3**	2	OP080803	*Neobacillus drentensis*	Firmicutes	99.5	desert soil
**LH3H4**	0	OP080804	*Pseudarthrobacter psychrotolerans*	Actinobacteria	99.3	Antarctic soil

Strains	No. of isolates	Genbank accession no.	Phylogenetically nearest taxon based on 16S rRNA	Phylum	Identity %	Sources of the nearest taxon
**LH3H5**	3	OP080805	*Brevundimonas basaltis*	Proteobacteria	97.9	black sand
**LH3H6**	2	OP080806	*Brevundimonas subvibrioides*	Proteobacteria	98.1	oligotrophic environments
**LH3H7**	1	OP080807	*Brevundimonas variabilis*	Proteobacteria	98.6	oligotrophic environments
**LH3H8**	2	OP080808	*Brevundimonas subvibrioides*	Proteobacteria	99.6	oligotrophic environments
**LH3H9**	2	OP080809	*Sphingomonas psychrolutea*	Proteobacteria	98.9	glacier ice
**LH3H10**	1	OP080810	*Nocardioides alpinus*	Actinobacteria	99.1	alpine glacier cryoconite
**LH3H11**	2	OP080811	*Bosea caraganae*	Proteobacteria	98.6	root nodules
**LH3H12**	3	OP080812	*Streptomyces pratensis*	Actinobacteria	99.5	grassy fields
**LH3H13**	2	OP080813	*Streptomyces pratensis*	Actinobacteria	99.5	grassy fields
**LH3H14**	1	OP080814	*Hymenobacter yonginensis*	Bacteroidetes	97.2	rtificial lake
**LH3H15**	2	OP080815	*Phenylobacterium panacis*	Proteobacteria	96.9	the rhizosphere of rusty mountain ginseng
**LH3H16**	3	OP080816	*Nocardioides alpinus*	Actinobacteria	98.7	alpine glacier cryoconite
**LH3H17**	3	OP080817	*Phenylobacterium aquaticum*	Proteobacteria	97.1	tumors on stone fruit rootstocks
**LH3N1**	3	OP080818	*Nocardioides alpinus*	Actinobacteria	97.4	alpine glacier cryoconite
**LH3N2**	2	OP080819	*Arthrobacter ruber*	Actinobacteria	99.9	glacier ice
**LH3N3**	4	OP080820	*Pseudarthrobacter psychrotolerans*	Actinobacteria	99.3	Antarctic soil
**LH3N4**	4	OP080821	*Pseudarthrobacter psychrotolerans*	Actinobacteria	99.4	Antarctic soil
**LH3N5**	4	OP080822	*Nocardioides furvisabuli*	Actinobacteria	98.4	black sand
**LH3N6**	4	OP080823	*Nocardioides alpinus*	Actinobacteria	99.4	alpine glacier cryoconite
**LH3U1**	3	OP080824	*Micromonospora nickelidurans*	Actinobacteria	98.0	nickel-mining site
**LH3U2**	1	OP080825	*Nocardioides szechwanensis*	Actinobacteria	99.2	glacier
**LH3U3**	4	OP080826	*Peribacillus muralis*	Firmicutes	97.2	Soil
**LH3U4**	2	OP080827	*Nocardioides zhouii*	Actinobacteria	98.3	Hailuogou glacier
**LH3U5**	2	OP080828	*Nocardioides zhouii*	Actinobacteria	98.7	Hailuogou glacier
**LH3U6**	3	OP080829	*Erythrobacter ramosus*	Proteobacteria	99.2	marine cyanobacterial mat
**LH3U7**	1	OP080830	*Brevundimonas basaltis*	Proteobacteria	97.9	black sand
**LH3U8**	2	OP080831	*Sphingomonas fonticola*	Proteobacteria	96.8	spring water
**LH3U9**	2	OP080832	*Nocardioides alpinus*	Actinobacteria	97.8	alpine glacier cryoconite
**LH3U10**	3	OP080833	*Erythrobacter ramosus*	Proteobacteria	98.7	marine cyanobacterial mat
**LH4H1**	2	OP080834	*Modestobacter caceresii*	Actinobacteria	99.2	stem tissues of rice,
**LH4H2**	2	OP080835	*Cellulomonas xylanilytica*	Actinobacteria	99.1	decayed elmtree
**LH4H3**	3	OP080836	*Modestobacter versicolor*	Actinobacteria	97.8	biological soil crusts
**LH4H4**	3	OP080837	*Dankookia rubra*	Proteobacteria	98.1	sediment of a shallow stream
**LH4H5**	3	OP080838	*Blastococcus aggregatus*	Actinobacteria	98.4	surface of marble and calcareous stones
**LH4H6**	1	OP080839	*Mycetocola zhujimingii*	Actinobacteria	98.6	faeces of Tibetan antelopes
**LH4H7**	1	OP080840	*Dankookia rubra*	Proteobacteria	98.4	sediment of a shallow stream
**LH4H8**	3	OP080841	*Nocardioides endophyticus*	Actinobacteria	98.9	herbaceous plant roots
**LH4H9**	2	OP080842	*Dankookia rubra*	Proteobacteria	98.7	sediment of a shallow stream
**LH4H10**	2	OP080843	*Modestobacter versicolor*	Actinobacteria	98.9	biological soil crusts
**LH4H11**	3	OP080844	*Roseomonas aquatica*	Proteobacteria	99.3	drinking water

Strains	No. of isolates	Genbank accession no.	Phylogenetically nearest taxon based on 16S rRNA	Phylum	Identity %	Sources of the nearest taxon
**LH4H12**	2	OP080845	*Streptomyces cavourensis*	Actinobacteria	99.2	soil
**LH4N1**	3	OP080846	*Pseudarthrobacter psychrotolerans*	Actinobacteria	99.3	Antarctic soil
**LH4N2**	3	OP080847	*Cryobacterium levicorallinum*	Actinobacteria	99.4	ice
**LH4N3**	1	OP080848	*Sphingomonas kaistensis*	Proteobacteria	98.5	soil in South Korea
**LH4N4**	3	OP080849	*Sphingomonas olei*	Proteobacteria	98.2	oil-contaminated soil
**LH4N5**	3	OP080850	*Pseudonocardia carboxydivorans*	Actinobacteria	99.4	pollutant soil
**LH4N6**	3	OP080851	*Pararhizobium herbae*	Proteobacteria	99.3	soil
**LH4N7**	2	OP080852	*Sphingorhabdus planktonica*	Proteobacteria	98.6	oligotrophic freshwater lake in Bavaria, Germany
**LH4N8**	3	OP080853	*Modestobacter caceresii*	Actinobacteria	99.4	stem tissues of rice,
**LH4N9**	3	OP080854	*Dankookia rubra*	Proteobacteria	98.3	sediment of a shallow stream
**LH4U1**	2	OP080855	*Micromonospora zamorensis*	Actinobacteria	99.4	rhizosphere of Pisum sativum
**LH4U2**	1	OP080856	*Modestobacter versicolor*	Actinobacteria	99.3	biological soil crusts
**LH4U3**	1	OP080857	*Roseomonas aquatica*	Proteobacteria	99.3	drinking water
**LH4U5**	0	OP080858	*Hymenobacter elongatus*	Bacteroidetes	99.4	Victoria Upper Glacier
**LH4U6**	0	OP080859	*Modestobacter versicolor*	Actinobacteria	98.7	biological soil crusts
**LH4U7**	2	OP080860	*Belnapia rosea*	Proteobacteria	98.8	soil
**LH4U8**	2	OP080861	*Micromonospora arida*	Actinobacteria	99.4	extreme hyper-arid Atacama Desert soil
**LH4U9**	1	OP080862	*Sphingomonas kaistensis*	Proteobacteria	98.4	soil in South Korea
**LH4U10**	2	OP080863	*Modestobacter versicolor*	Actinobacteria	98.8	biological soil crusts
**LH4U11**	1	OP080864	*Noviherbaspirillum psychrotolerans*	Proteobacteria	98.2	oil-contaminated soil
**LH4U12**	3	OP080865	*Nocardioides szechwanensis*	Actinobacteria	99.2	glacier
**LH4U13**	2	OP080866	*Spirosoma rigui*	Bacteroidetes	97.0	Apple Orchard Soil
**LH4U14**	1	OP080867	*Pseudarthrobacter psychrotolerans*	Actinobacteria	99.3	Antarctic soil
**LH4U15**	1	OP080868	*Microbacterium hatanonis*	Actinobacteria	98.8	contaminant of hairspray
**LH4U16**	1	OP080869	*Nocardioides astragali*	Actinobacteria	98.1	nodule of wild Astragalus chrysopterus in northwestern China
**LH4U17**	1	OP080870	*Sphingomonas panaciterrae*	Proteobacteria	98.6	oil of a ginseng field
**LH5H1**	2	OP080871	*Pseudarthrobacter psychrotolerans*	Actinobacteria	99.0	Antarctic soil
**LH5H2**	2	OP080872	*Sphingomonas olei*	Proteobacteria	98.3	oil-contaminated soil
**LH5H3**	3	OP080873	*Nocardioides furvisabuli*	Actinobacteria	98.1	black sand
**LH5H4**	3	OP080874	*Brevundimonas variabilis*	Proteobacteria	98.8	oligotrophic environments
**LH5H5**	3	OP080875	*Nocardioides furvisabuli*	Actinobacteria	98.2	black sand
**LH5H6**	3	OP080876	*Brevundimonas variabilis*	Proteobacteria	99.0	oligotrophic environments
**LH5H7**	1	OP080877	*Blastococcus aggregatus*	Actinobacteria	99.2	surface of marble and calcareous stones
**LH5H8**	1	OP080878	*Noviherbaspirillum psychrotolerans*	Proteobacteria	98.3	oil-contaminated soil
**LH5H9**	3	OP080879	*Deinococcus wulumuqiensis*	Deinococcus	99.1	radiation-polluted soil

Strains	No. of isolates	Genbank accession no.	Phylogenetically nearest taxon based on 16S rRNA	Phylum	Identity %	Sources of the nearest taxon
**LH5H10**	1	OP080880	*Noviherbaspirillum psychrotolerans*	Proteobacteria	98.5	oil-contaminated soil
**LH5H11**	2	OP080881	*Streptomyces cavourensis*	Actinobacteria	99.2	marine fungal
**LH5H12**	2	OP080882	*Agreia pratensis*	Actinobacteria	99.2	phyllosphere of grasses
**LH5H13**	3	OP080883	*Modestobacter versicolor*	Actinobacteria	98.9	biological soil crusts
**LH5H14**	1	OP080884	*Micromonospora vinacea*	Actinobacteria	98.9	Pisum sativum nodules
**LH5H15**	3	OP080885	*Rhodococcus sovatensis*	Actinobacteria	99.6	the hypersaline and heliothermal Lake Ursu
**LH5N1**	4	OP080886	*Agreia pratensis*	Actinobacteria	98.8	phyllosphere of grasses
**LH5N2**	4	OP080887	*Pseudarthrobacter psychrotolerans*	Actinobacteria	98.9	Antarctic soil
**LH5N3**	4	OP080888	*Paeniglutamicibacter sulfureus*	Actinobacteria	98.8	soil
**LH5N4**	4	OP080889	*Pseudarthrobacter psychrotolerans*	Actinobacteria	99.1	Antarctic soil
**LH5N5**	4	OP080890	*Sphingomonas panaciterrae*	Proteobacteria	98.2	oil of a ginseng field
**LH5N6**	14	OP080891	*Pseudarthrobacter sulfonivorans*	Actinobacteria	99.1	pollutant soil
**LH5N7**	4	OP080892	*Agreia pratensis*	Actinobacteria	98.9	phyllosphere of grasses
**LH5N8**	4	OP080893	*Micrococcus endophyticus*	Actinobacteria	98.7	surface-sterilized Aquilaria sinensis roots
**LH5N9**	4	OP080894	*Hymenobacter elongatus*	Bacteroidetes	99.2	Victoria Upper Glacier
**LH5U1**	2	OP080895	*Microvirga aerilata*	Proteobacteria	98.1	air
**LH5U2**	1	OP080896	*Blastococcus litoris*	Actinobacteria	98.0	soil
**LH5U4**	1	OP080897	*Hymenobacter elongatus*	Bacteroidetes	99.1	Victoria Upper Glacier
**LH5U5**	1	OP080898	*Noviherbaspirillum psychrotolerans*	Proteobacteria	98.2	oil-contaminated soil
**LH5U6**	1	OP080899	*Noviherbaspirillum psychrotolerans*	Proteobacteria	98.2	oil-contaminated soil
**LH5U7**	2	OP080900	*Micromonospora zamorensis*	Actinobacteria	99.3	rhizosphere of Pisum sativum
**LH5U8**	2	OP080901	*Pseudarthrobacter psychrotolerans*	Actinobacteria	99.0	Antarctic soil
**LH5U10**	0	OP080902	*Arthrobacter ruber*	Actinobacteria	99.7	glacier ice
**LH5U11**	2	OP080903	*Arthrobacter zhaoguopingii*	Actinobacteria	98.8	plateau wildlife on the Qinghai-Tibet Plateau of PR China
**LH5U12**	3	OP080904	*Microbacterium pumilum*	Actinobacteria	99.6	soil
**LH5U13**	3	OP080905	*Noviherbaspirillum psychrotolerans*	Proteobacteria	98.2	oil-contaminated soil
**LL1H1**	1	OP080906	*Pseudarthrobacter psychrotolerans*	Actinobacteria	99.1	Antarctic soil
**LL1H2**	1	OP080907	*Pseudarthrobacter psychrotolerans*	Actinobacteria	99.1	Antarctic soil
**LL1H3**	1	OP080908	*Arthrobacter zhaoguopingii*	Actinobacteria	99.0	plateau wildlife on the Qinghai-Tibet Plateau of PR China
**LL1H6**	1	OP080909	*Clavibacter michiganensis*	Actinobacteria	99.7	bean seeds (Phaseolus vulgaris L.)

Strains	No. of isolates	Genbank accession no.	Phylogenetically nearest taxon based on 16S rRNA	Phylum	Identity %	Sources of the nearest taxon
**LL1H7**	1	OP080910	*Deinococcus marmoris*	Deinococcus-Thermus	98.9	continental Antarctica
**LL1H8**	1	OP080911	*Pseudarthrobacter psychrotolerans*	Actinobacteria	99.1	Antarctic soil
**LL1H9**	1	OP080912	*Pseudarthrobacter psychrotolerans*	Actinobacteria	99.1	Antarctic soil
**LL1N1**	1	OP080913	*Knoellia locipacati*	Actinobacteria	98.5	soil of the Demilitarized Zone in South Korea
**LL1N2**	1	OP080914	*Pseudarthrobacter psychrotolerans*	Actinobacteria	95.2	Antarctic soil
**LL1N3**	1	OP080915	*Brevundimonas alba*	Proteobacteria	98.2	oligotrophic environments
**LL1N4**	1	OP080916	*Massilia niabensis*	Proteobacteria	98.6	air samples
**LL1N5**	1	OP080917	*Noviherbaspirillum psychrotolerans*	Proteobacteria	98.2	oil-contaminated soil
**LL1N6**	1	OP080918	*Pseudarthrobacter psychrotolerans*	Actinobacteria	98.9	Antarctic soil
**LL1N7**	1	OP080919	*Massilia niabensis*	Proteobacteria	98.6	air samples
**LL1N8**	1	OP080920	*Pseudarthrobacter psychrotolerans*	Actinobacteria	99.1	Antarctic soil
**LL1N9**	1	OP080921	*Arthrobacter zhaoguopingii*	Actinobacteria	98.7	plateau wildlife on the Qinghai-Tibet Plateau of PR China
**LL1N11**	1	OP080922	*Massilia niabensis*	Proteobacteria	98.6	air samples
**LL1N12**	1	OP080923	*Arthrobacter ruber*	Actinobacteria	98.3	glacier ice
**LL1N13**	1	OP080924	*Pseudarthrobacter psychrotolerans*	Actinobacteria	99.1	Antarctic soil
**LL1N14**	1	OP080925	*Brevundimonas alba*	Proteobacteria	98.6	oligotrophic environments
**LL1N15**	1	OP080926	*Nocardioides furvisabuli*	Actinobacteria	98.4	black sand
**LL1N17**	1	OP080927	*Larkinella rosea*	Bacteroidetes	98.4	manganese mine soil
**LL1N18**	1	OP080928	*Cryobacterium breve*	Actinobacteria	99.1	glacier
**LL1N19**	1	OP080929	*Arthrobacter zhaoguopingii*	Actinobacteria	98.9	plateau wildlife on the Qinghai-Tibet Plateau of PR China
**LL1N20**	1	OP080930	*Nocardioides furvisabuli*	Actinobacteria	98.3	black sand
**LL2H1**	1	OP080931	*Brevundimonas vesicularis*	Proteobacteria	98.5	Leech
**LL2H2**	1	OP080932	*Brevundimonas albigilva*	Proteobacteria	98.2	forest soil
**LL2H3**	1	OP080933	*Acidovorax soli*	Proteobacteria	98.7	landfill soil
**LL2H5**	1	OP080934	*Sphingomonas melonis*	Proteobacteria	98.7	yellow Spanish melon fruits
**LL2H6**	1	OP080935	*Acidovorax soli*	Proteobacteria	98.1	landfill soil
**LL2H7**	1	OP080936	*Methylobacterium oryzae*	Proteobacteria	98.8	stem tissues of rice,
**LL2N1**	3	OP080937	*Adhaeribacter pallidiroseus*	Bacteroidetes	96.6	birch tree
**LL2N2**	1	OP080938	*Adhaeribacter aerolatus*	Bacteroidetes	94.0	air
**LL2N4**	1	OP080939	*Methylobacterium oryzae*	Proteobacteria	99.5	stem tissues of rice,
**LL2N6**	1	OP080940	*Acidovorax soli*	Proteobacteria	98.7	landfill soil
**LL2N7**	1	OP080941	*Sphingomonas melonis*	Proteobacteria	99.3	yellow Spanish melon fruits

Strains	No. of isolates	Genbank accession no.	Phylogenetically nearest taxon based on 16S rRNA	Phylum	Identity %	Sources of the nearest taxon
**LL2U4**	1	OP080942	*Hymenobacter lapidarius*	Bacteroidetes	96.6	rocks in Antarctica
**LL2U6**	1	OP080943	*Methylobacterium iners*	Proteobacteria	98.7	air
**LL3H1**	1	OP080944	*Pseudarthrobacter psychrotolerans*	Actinobacteria	99.3	Antarctic soil
**LL3H2**	1	OP080945	*Brevundimonas staleyi*	Proteobacteria	98.4	activated sludge
**LL3H3**	1	OP080946	*Cryobacterium breve*	Actinobacteria	99.7	glacier
**LL3H5**	1	OP080947	*Pseudarthrobacter psychrotolerans*	Actinobacteria	99.0	Antarctic soil
**LL3H6**	0	OP080948	*Cryobacterium arcticum*	Actinobacteria	99.5	forest soil
**LL3H9**	1	OP080949	*Massilia eurypsychrophila*	Proteobacteria	98.4	ice core
**LL3N1**	1	OP080950	*Pseudarthrobacter psychrotolerans*	Actinobacteria	98.8	Antarctic soil
**LL3N2**	1	OP080951	*Brevundimonas mongoliensis*	Proteobacteria	97.9	Oil-Contaminated Soil
**LL3N3**	1	OP080952	*Pseudarthrobacter psychrotolerans*	Actinobacteria	98.8	Antarctic soil
**LL3N4**	1	OP080953	*Pseudarthrobacter psychrotolerans*	Actinobacteria	98.8	Antarctic soil
**LL3N5**	1	OP080954	*Brevundimonas basaltis*	Proteobacteria	98.4	black sand
**LL3N6**	1	OP080955	*Pseudarthrobacter psychrotolerans*	Actinobacteria	98.8	Antarctic soil
**LL3N7**	1	OP080956	*Cryobacterium arcticum*	Actinobacteria	99.5	forest soil
**LL3U1**	1	OP080957	*Pseudarthrobacter psychrotolerans*	Actinobacteria	98.8	Antarctic soil
**LL4H2**	1	OP080958	*Agreia pratensis*	Actinobacteria	98.7	phyllosphere of grasses
**LL4H3**	1	OP080959	*Labedella populi*	Actinobacteria	99.1	plants in the Taklamakan Desert
**LL4H4**	1	OP080960	*Cryobacterium arcticum*	Actinobacteria	99.6	forest soil
**LL4H5**	1	OP080961	*Cryobacterium levicorallinum*	Actinobacteria	99.5	ice
**LL4H7**	1	OP080962	*Nocardioides psychrotolerans*	Actinobacteria	99.0	oil-contaminated site located in Kaohsiung county, Taiwan
**LL4H9**	1	OP080963	*Cryobacterium luteum*	Actinobacteria	99.7	meltwater
**LL4N1**	1	OP080964	*Arthrobacter ruber*	Actinobacteria	98.7	glacier ice
**LL4N2**	1	OP080965	*Cryobacterium levicorallinum*	Actinobacteria	99.4	ice
**LL4N4**	1	OP080966	*Phyllobacterium trifolii*	Proteobacteria	99.3	Spanish soils
**LL4N5**	1	OP080967	*Nocardioides psychrotolerans*	Actinobacteria	99.0	oil-contaminated site located in Kaohsiung county, Taiwan
**LL4N6**	1	OP080968	*Cryobacterium levicorallinum*	Actinobacteria	99.2	ice
**LL4N7**	1	OP080969	*Pseudarthrobacter psychrotolerans*	Actinobacteria	99.1	Antarctic soil
**LL4N8**	1	OP080970	*Polymorphobacter glacialis*	Proteobacteria	99.2	ice core
**LL4N9**	1	OP080971	*Hymenobacter elongatus*	Bacteroidetes	99.1	Victoria Upper Glacier
**LL4N10**	1	OP080972	*Mycetocola manganoxydans*	Actinobacteria	98.6	the Taklamakan desert
**LL4N12**	1	OP080973	*Brevundimonas subvibrioides*	Proteobacteria	98.4	oligotrophic environments

Strains	No. of isolates	Genbank accession no.	Phylogenetically nearest taxon based on 16S rRNA	Phylum	Identity %	Sources of the nearest taxon
**LL4U2**	1	OP080974	*Arthrobacter ruber*	Actinobacteria	99.0	glacier ice
**LL4U3**	1	OP080975	*Polaromonas glacialis*	Proteobacteria	99.0	ice core
**LL4U4**	1	OP080976	*Cellulomonas humilata*	Actinobacteria	99.0	soil
**LL4U5**	1	OP080977	*Modestobacter italicus*	Actinobacteria	99.3	a black patina of the surface of a Carrara marble blockin
**LL4U6**	1	OP080978	*Skermanella aerolata*	Proteobacteria	99.3	air
**LL4U7**	1	OP080979	*Skermanella aerolata*	Proteobacteria	99.4	air
**LL5H1**	1	OP080980	*Cryobacterium levicorallinum*	Actinobacteria	99.6	ice
**LL5H2**	1	OP080981	*Pseudarthrobacter psychrotolerans*	Actinobacteria	99.0	Antarctic soil
**LL5H4**	1	OP080982	*Arthrobacter ruber*	Actinobacteria	99.6	glacier ice
**LL5H5**	1	OP080983	*Marisediminicola antarctica*	Actinobacteria	99.1	Queen Maud Land
**LL5H6**	1	OP080984	*Cryobacterium levicorallinum*	Actinobacteria	99.6	ice
**LL5H8**	1	OP080985	*Pseudarthrobacter psychrotolerans*	Actinobacteria	99.3	Antarctic soil
**LL5H10**	1	OP080986	*Plantibacter flavus*	Actinobacteria	98.7	plant soil
**LL5N1**	1	OP080987	*Pseudarthrobacter psychrotolerans*	Actinobacteria	99.1	Antarctic soil
**LL5N2**	1	OP080988	*Cryobacterium levicorallinum*	Actinobacteria	99.6	ice
**LL5N3**	1	OP080989	*Marisediminicola antarctica*	Actinobacteria	99.3	Queen Maud Land
**LL5N4**	1	OP080990	*Massilia psychrophila*	Proteobacteria	98.5	ice core
**LL5N5**	1	OP080991	*Hymenobacter sedentarius*	Bacteroidetes	96.2	soil
**LL5N6**	1	OP080992	*Pseudarthrobacter psychrotolerans*	Actinobacteria	99.1	Antarctic soil
**LL5N7**	1	OP080993	*Flavobacterium omnivorum*	Bacteroidetes	98.4	China No. 1 glacier
**LL5N8**	1	OP080994	*Arthrobacter ruber*	Actinobacteria	99.6	glacier ice
**LL5U1**	1	OP080995	*Hymenobacter pedocola*	Bacteroidetes	96.3	soil
**LL5U2**	1	OP080996	*Arthrobacter ruber*	Actinobacteria	99.6	glacier ice
**LL5U3**	1	OP080997	*Hymenobacter elongatus*	Bacteroidetes	99.0	Victoria Upper Glacier
**LL5U4**	1	OP080998	*Massilia niabensis*	Proteobacteria	98.3	air samples
**LL5U5**	1	OP080999	*Aureimonas glaciei*	Proteobacteria	97.3	ice core
**LL5U6**	1	OP081000	*Pseudarthrobacter psychrotolerans*	Actinobacteria	99.2	Antarctic soil

Table S3. List of genera represented in the Venn diagram of Fig S1

Medium	T	N .	Shared Genera
**R2A**	HT	10	*Cellulomonas, Marmoricola, Mycetocola, Rhodococcus, Spirosoma, Deinococcus, Peribacillus, Acidovorax, Rudaea, Pseudonocardia*
**PYGV**	HT	7	*Cryobacterium, Longivirga, Promicromonospora, Neobacillus Bosea, Microvirga, Sphingorhabdus*
**R2A, PYGV**	HT	9	*Blastococcus, Geodermatophilus, Microbacterium, Bacillus Erythrobacter, Phenylobacterium, Roseomonas, Agreia, Hymenobacter*
**TSB**	HT	2	*Paeniglutamicibacter, Caulobacter*
**TSB, R2A**	HT	1	*Clavibacter*
**TSB, PYGV**	HT	0	/
**TSB, R2A, PYGV**	HT	0	/
**TSB 0.1X**	HT	2	*Belnapia, Pararhizobium*
**TSB 0.1X, R2A**	HT	0	/
**TSB 0.1X, PYGV**	HT	0	/
**TSB 0.1X, R2A, PYGV**	HT	3	*Mycolicibacterium, Arthrobacter, Methylobacterium*
**TSB 0.1X, TSB**	HT	0	/
**TSB 0.1X, R2A, TSB**	HT	2	*Streptomyces, Dankookia*
**TSB 0.1X, TSB, PYGV**	HT	0	/
**R2A, PYGV, TSB, TSB 0.1X**	HT	8	*Micrococcus, Micromonospora, Modestobacter, Brevundimonas Noviherbaspirillum, Nocardioides, Pseudarthrobacter, Sphingomonas*
**R2A**	LT	11	*Methylobacterium, Agreia, Clavibacter, Mycetocola, Knoellia, Labedella, Larkinella, Aureimonas, Phyllobacterium, Polaromonas, Polymorphobacter*
**PYGV**	LT	5	*Skermanella, Cellulomonas, Modestobacter, Flavobacterium, Deinococcus*
**R2A, PYGV**	LT	4	*Hymenobacter, Marisediminicola, Adhaeribacter, Sphingomonas*
**TSB**	LT	1	*Noviherbaspirillum*
**TSB, R2A**	LT	0	/
**TSB, PYGV**	LT	0	/
**TSB, R2A, PYGV**	LT	1	*Acidovorax*
**TSB 0.1X**	LT	1	*Plantibacter*
**TSB 0.1X, R2A**	LT	0	/
**TSB 0.1X, PYGV**	LT	0	/
**TSB 0.1X, R2A, PYGV**	LT	4	*Cryobacterium, Brevundimonas, Massilia, Nocardioides*
**TSB 0.1X, TSB**	LT	0	/
**TSB 0.1X, R2A, TSB**	LT	0	/
**TSB 0.1X, TSB, PYGV**	LT	0	/
**R2A, PYGV, TSB, TSB 0.1X**	LT	2	*Pseudarthrobacter, Arthrobacter*

HT and LT represented the high culture temperature and low culture temperature.

Table S4. List of genera represented in the Venn diagram of Fig 5(a) – (f)

Medium	P.	N.	Shared Genera
**R2A**	H&N	0	/
**PYGV**	H&N	0	/
**R2A, PYGV**	H&N	2	*Streptomyces, Sphingorhabdus*
**TSB**	H&N	0	/
**TSB, R2A**	H&N	0	/
**TSB, PYGV**	H&N	0	/
**TSB, R2A, PYGV**	H&N	0	/
**TSB 0.1x**	H&N	0	/
**TSB 0.1x, R2A**	H&N	1	*Rudaea*
**TSB 0.1x, PYGV**	H&N	1	*Longivirga*
**TSB 0.1x, R2A, PYGV**	H&N	3	*Cryobacterium, Pseudonocardia, Pararhizobium*
**TSB 0.1x, TSB**	H&N	0	/
**TSB 0.1x, R2A, TSB**	H&N	2	*Modestobacter, Dankookia*
**TSB 0.1x, PYGV, TSB**	H&N	1	*Caulobacter*
**TSB 0.1x, R2A, PYGV, TSB**	H&N	13	*Agreia, Arthrobacter, Micrococcus, Mycolicibacterium, Nocardioides, Paeniglutamicibacter, Pseudarthrobacter, Hymenobacter, Acidovorax, Brevundimonas, Methylobacterium, Noviherbaspirillum, Sphingomonas*
**R2A**	L&N	7	*Knoellia, Larkinella, Methylobacterium, Phyllobacterium, Polymorphobacter, Hymenobacter, Mycetocola*
**PYGV**	L&N	3	*Sphingomonas, Marisediminicola, Flavobacterium*
**R2A, PYGV**	L&N	0	/
**TSB**	L&N	2	*Noviherbaspirillum, Acidovorax*
**TSB, R2A**	L&N	0	/
**TSB, PYGV**	L&N	0	/
**TSB, R2A, PYGV**	L&N	0	/
**TSB 0.1x**	L&N	0	/
**TSB 0.1x, R2A**	L&N	0	/
**TSB 0.1x, PYGV**	L&N	0	/
**TSB 0.1x, R2A, PYGV**	L&N	4	*Massilia, Brevundimonas, Cryobacterium, Nocardioides*
**TSB 0.1x, TSB**	L&N	0	/
**TSB 0.1x, R2A, TSB**	L&N	0	/
**TSB 0.1x, PYGV, TSB**	L&N	0	/
**TSB 0.1x, R2A, PYGV, TSB**	L&N	3	*Pseudarthrobacter, Arthrobacter, Adhaeribacter*
**R2A**	H&O	3	*Mycetocola, Hymenobacter, Noviherbaspirillum*
**PYGV**	H&O	0	/
**R2A, PYGV**	H&O	1	*Microbacterium, Bosea*
**TSB**	H&O	0	/
**TSB, R2A**	H&O	1	*Cellulomonas*
**TSB, PYGV**	H&O	0	/
**TSB, R2A, PYGV**	H&O	0	/

Medium	P.	N.	Shared Genera
**TSB 0.1x**	H&O	0	/
**TSB 0.1x, R2A**	H&O	1	*Agreia*
**TSB 0.1x, PYGV**	H&O	3	*Agreia, Streptomyces, Neobacillus*
**TSB 0.1x, R2A, PYGV**	H&O	8	*Arthrobacter, Micromonospora, Mycolicibacterium, Pseudarthrobacter, Deinococcus, Bacillus, Brevundimonas, Phenylobacterium*
**TSB 0.1x, TSB**	H&O	0	/
**TSB 0.1x, R2A, TSB**	H&O	2	*Rhodococcus, Roseomonas*
**TSB 0.1x, PYGV, TSB**	H&O	1	*Methylobacterium*
**TSB 0.1x, R2A, PYGV, TSB**	H&O	7	*Blastococcus, Clavibacter, Marmoricola, Modestobacter, Nocardioides, Dankookia, Sphingomonas*
**R2A**	L&O	7	*Sphingomonas, Methylobacterium, Massilia, Marisediminicola, Labedella, Clavibacter, Agreia*
**PYGV**	L&O	2	*Deinococcus, Nocardioides*
**R2A, PYGV**	L&O	2	*Arthrobacter, Acidovorax*
**TSB**	L&O	0	/
**TSB, R2A**	L&O	0	/
**TSB, PYGV**	L&O	0	/
**TSB, R2A, PYGV**	L&O	0	/
**TSB 0.1x**	L&O	1	*Plantibacter*
**TSB 0.1x, R2A**	L&O	0	/
**TSB 0.1x, PYGV**	L&O	0	/
**TSB 0.1x, R2A, PYGV**	L&O	2	*Cryobacterium, Brevundimonas*
**TSB 0.1x, TSB**	L&O	0	/
**TSB 0.1x, R2A, TSB**	L&O	0	/
**TSB 0.1x, PYGV, TSB**	L&O	0	/
**TSB 0.1x, R2A, PYGV, TSB**	L&O	1	*Pseudarthrobacter*
**R2A**	H&R	0	/
**PYGV**	H&R	5	*Bacillus, Blastococcus, Brevundimonas, Hymenobacter, Roseomonas*
**R2A, PYGV**	H&R	6	*Microvirga, Modestobacter, Promicromonospora, Pseudarthrobacter, Sphingomonas, Spirosoma*
**TSB**	H&R	0	/
**TSB, R2A**	H&R	0	/
**TSB, PYGV**	H&R	0	/
**TSB, R2A, PYGV**	H&R	1	*Peribacillus*
**TSB 0.1x**	H&R	0	/
**TSB 0.1x, R2A**	H&R	0	/
**TSB 0.1x, PYGV**	H&R	0	*Belnapia*
**TSB 0.1x, R2A, PYGV**	H&R	5	*Arthrobacter, Geodermatophilus, Microbacterium, Nocardioides, Noviherbaspirillum*
**TSB 0.1x, TSB**	H&R	0	/
**TSB 0.1x, R2A, TSB**	H&R	0	/
**TSB 0.1x, PYGV, TSB**	H&R	0	/
**TSB 0.1x, R2A, PYGV, TSB**	H&R	2	*Erythrobacter, Micromonospora*

**R2A**	L&R	3	*Methylobacterium, Polaromonas, Aureimonas*
**PYGV**	L&R	5	*Hymenobacter, Cellulomonas, Modestobacter, Skermanella, Massilia*
**R2A, PYGV**	L&R	2	*Pseudarthrobacter, Arthrobacter*

N, O and R represented the pretreatment of normal, oxidation and radiation respectively.

H and L represented the high culture temperature and low culture temperature.

Table S5. List of genera represented in the Venn diagram of Fig Fig 5(g) – (h)

**Mediun**	**T**	**N .**	**Unshared Genera**
**R2A-O**	HT	2	Mycetocola,Pseudonocardia
**PYGV-O**	HT	1	Sphingorhabdus
**R2A-O**	LT	5	Agreia,Clavibacter,Sphingomonas,Marisediminicola,Labedella
**PYGV-O**	LT	2	Nocardioides,Deinococcus
**TSB 0.1x-O**	LT	1	Plantibacter
**R2A-R**	LT	2	Aureimonas,Polaromonas
**PYGV-R**	LT	4	Hymenobacter,Modestobacter,Skermanella,Cellulomonas

HT and LT represented the high culture temperature and low culture temperature.
